# Structural analysis of an endogenous 4-megadalton succinyl-CoA-generating metabolon

**DOI:** 10.1038/s42003-023-04885-0

**Published:** 2023-05-22

**Authors:** Ioannis Skalidis, Fotis L. Kyrilis, Christian Tüting, Farzad Hamdi, Toni K. Träger, Jaydeep Belapure, Gerd Hause, Marta Fratini, Francis J. O’Reilly, Ingo Heilmann, Juri Rappsilber, Panagiotis L. Kastritis

**Affiliations:** 1 Interdisciplinary Research Center HALOmem, Charles Tanford Protein Center, Martin Luther University Halle-Wittenberg, Kurt-Mothes-Straße 3a, 06120 Halle/Saale, Germany; 2 Institute of Biochemistry and Biotechnology, Martin Luther University Halle-Wittenberg, Kurt-Mothes-Straße 3, 06120 Halle/Saale, Germany; 3 Biozentrum, Martin Luther University Halle-Wittenberg, Weinbergweg 22, 06120 Halle/Saale, Germany; 4 Department of Plant Biochemistry, Institute of Biochemistry and Biotechnology, Martin Luther University Halle-Wittenberg, Kurt-Mothes-Str. 3a, 06120 Halle/Saale, Germany; 5 Center for Structural Biology, Center for Cancer Research, National Cancer Institute (NCI), Frederick, MD 21702-1201 USA; 6 Bioanalytics, Institute of Biotechnology, Technische Universität Berlin, 13355 Berlin, Germany; 7 Wellcome Centre for Cell Biology, School of Biological Sciences, University of Edinburgh, Edinburgh, EH9 3BF Scotland United Kingdom; 8 Institute of Chemical Biology, National Hellenic Research Foundation, Athens, 11635 Greece

**Keywords:** Cryoelectron microscopy, Cryoelectron microscopy, Enzymes, Biochemical networks

## Abstract

The oxoglutarate dehydrogenase complex (OGDHc) participates in the tricarboxylic acid cycle and, in a multi-step reaction, decarboxylates α-ketoglutarate, transfers succinyl to CoA, and reduces NAD+. Due to its pivotal role in metabolism, OGDHc enzymatic components have been studied in isolation; however, their interactions within the endogenous OGDHc remain elusive. Here, we discern the organization of a thermophilic, eukaryotic, native OGDHc in its active state. By combining biochemical, biophysical, and bioinformatic methods, we resolve its composition, 3D architecture, and molecular function at 3.35 Å resolution. We further report the high-resolution cryo-EM structure of the OGDHc core (E2o), which displays various structural adaptations. These include hydrogen bonding patterns confining interactions of OGDHc participating enzymes (E1o-E2o-E3), electrostatic tunneling that drives inter-subunit communication, and the presence of a flexible subunit (E3BPo), connecting E2o and E3. This multi-scale analysis of a succinyl-CoA-producing native cell extract provides a blueprint for structure-function studies of complex mixtures of medical and biotechnological value.

## Abstract (web summary)

Multi-scale and integrative structural analyses provide insight into metabolic regulation by the higher-order architecture of the *Chaetomium thermophilum* oxoglutarate dehydrogenase complex (OGDHc) metabolon.

## Introduction

The oxoglutarate dehydrogenase complex (OGDHc) is one of the main regulators of metabolic flux in the tricarboxylic cycle (TCA), meaning that cells maintain OGDHc abundance and activity under tight control^1,2^. In eukaryotes, OGDHc is active in the mitochondrion, but a fraction of the complex also localizes in the nucleus performing lysine succinylation of histones^3^. The OGDHc-catalyzed reaction – the decarboxylation of oxoglutarate (α-ketoglutarate) to succinyl-CoA – is important for energy production, the metabolic interaction between mitochondria and cytoplasm as well as the metabolism of neurotransmitters. This is because oxoglutarate (α-ketoglutarate) is generated in the TCA cycle during the oxidation of carbohydrates and fatty acids and by glutamate dehydrogenase during the oxidative deamination of glutamate. It is additionally produced by glutamate transamination as part of the malate–aspartate shuttle, which transfers reducing equivalents from the cytoplasm to mitochondria. This crucial position of OGDHc in metabolism and the association between decreased OGDHc activity in the brain and neurodegeneration has stimulated extensive research^4,5^. Furthermore, OGDHc is highly sensitive to reactive oxygen species (ROS)^6^, and its possible inhibition due to oxidative stress could prove detrimental to a cell’s overall metabolism. Such cancer-associated cellular conditions may impact OGDHc activity^7^, especially considering that oxoglutarate is an effector of p53-mediated tumor suppression^8^. Consequently, understanding structure-function relationships of OGDHc is of particular interest.

**Fig. 1:**
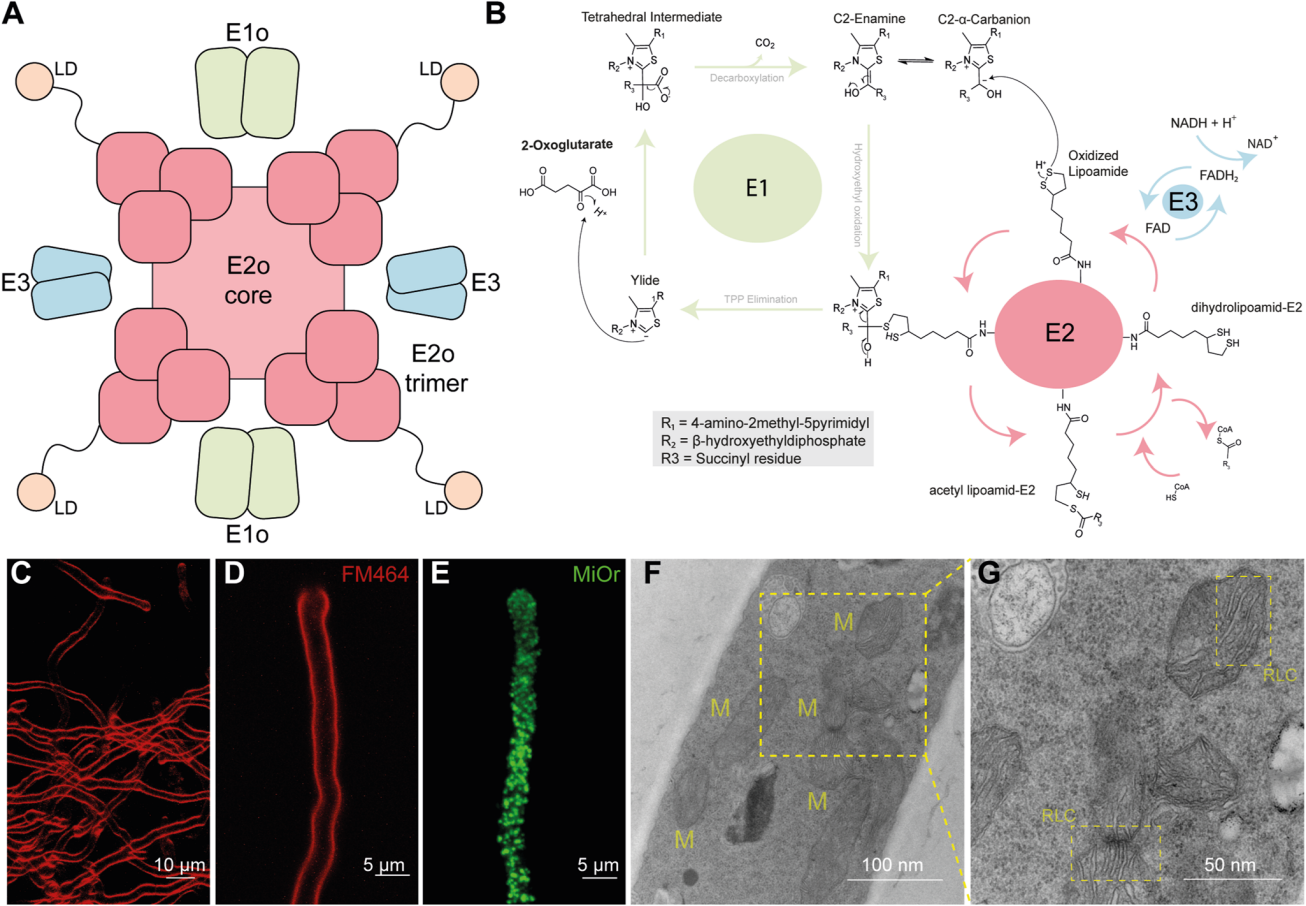
Previous knowledge on OGDHc and a morphological study of *C. thermophilum*, the model organism that is used to expand it. **A** Schematic representation of previous knowledge on OGDHc overall architecture. A cubic core comprised of 24 E2o proteins, with extended flexible linkers ending in the ordered LD domain, and E1o and E3 dimers on the periphery. **B** Complete reaction scheme of OGDHc. **C** Fluorescent light microscopy of isolated *C. thermophilum* filaments, with the cell membrane in red color, stained with FM464. **D** Fluorescent light microscopy of a single *C. thermophilum* filament, with the cell membrane in red color, stained with FM464. **E** Fluorescent light microscopy of a single *C. thermophilum* filament, with the highly abundant mitochondrial content visible in green color, stained with MiOr. **F** Transmission electron microscopy image of cryo-fixated ultra-thin sections of *C. thermophilum* filament. With “M” the mitochondria are annotated in the image. **G** Zoom-in into the mitochondria ultrastructures visible in **F**. The ribbon-like christae of the *C. thermophilum* mitochondria are visible (annotated as “RLC”).

At the molecular level, OGDHc is a megadalton-size assembly of multiple copies of three enzymes, namely E1o (oxoglutarate dehydrogenase, EC 1.2.4.2), E2o (dihydrolipoyl succinyltransferase, EC 2.3.1.61) and E3 (dihydrolipoyl dehydrogenase, EC 1.8.1.4). E2o forms the core of the complex with a stable stoichiometry of 24 copies in a cubic assembly, whereas the E1o and E3 proteins assemble on its periphery to carry out the complete reaction in a yet stoichiometrically and structurally unknown high-order metabolon (Fig. 1A). Although OGDHc single enzyme components have been structurally resolved and kinetic characterization of its discrete steps are studied^9–11^ (Fig. 1B), their intercommunication is unknown, hampering, therefore, structure-function studies underlying this metabolon. Due to the challenge of probing its endogenous structure and function, modern mass spectrometry analysis combined with cross-linking-based modeling was recently employed to derive insights into subunit proximity of OGDHc at low resolution without probing its activity^12^. This is due to the fact that the metabolon has not yet been reconstituted in vitro as a functional entity despite decades of research. In addition, the lipoyl domain (LD) that transfers lipoyl intermediates from one active site to the next (E1o → E2o → E3) is part of a highly flexible E2o arm, representing a particular challenge for its study by traditional structural methods. The potential involvement of additional structural elements, such as subunits that could contribute to OGDHc cross-communication, i.e., KGD4^13^, further complicates the complex’s characterization.

In this work, we endeavor to elucidate the structure of the OGDHc metabolon in the context of a native lysate fraction derived from *Chaetomium thermophilum* after a single-step fractionation of crude lysate (Supplementary Fig. 1). *C. thermophilum* is a thermophilic, filamentous fungus with an optimal growth temperature of 54 °C and the inherent thermostability of its proteins and protein complexes makes it an ideal model for structural studies focusing on multi-component targets, such as OGDHc or other protein complexes^14–16^. The inherent advantages of a thermophilic cell extract are put forth while we dissect its composition and architecture. We employ biochemical assays to reveal the complex’s kinetic parameters for producing succinyl-CoA, identify all its components, and utilize cryo-electron microscopy, crosslinking, and artificial intelligence (AI) to elucidate the complex’s mega-structure at high resolution with rigorous data collection strategies. Overall, we provide a multi-scale snapshot of a cell extract enriched in OGDHc that is underlined by flexible regions dictating transient enzyme-enzyme interactions, enzyme clusters that electrostatically channel OGDHc intermediates, and a higher-order architecture of OGDHc critical to the complex’s overall function.

## Results

### Biochemical isolation of an active cell extract from a thermophilic, filamentous fungus

**Fig. 2:**
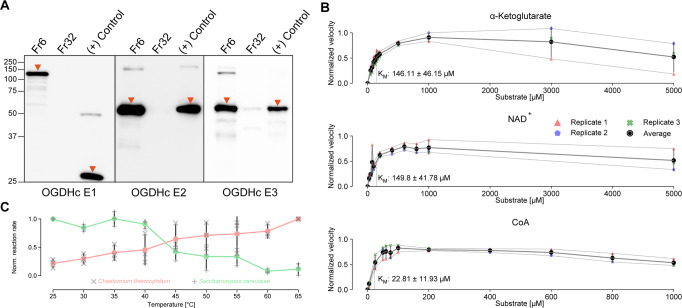
Biochemical characterization of OGDHc. **A** Western blots displaying the detection of all OGDHc components in the native cell extract fraction at the expected molecular weight (MW). A low-MW fraction was used as negative control, whereas the overexpressed protein that was used to create the antibodies was employed as a positive control. Due to the large size of the E1o protein, a fragment was employed for this reason (see Methods). Corresponding bands have been annotated with an orange arrow. **B** Enzymatic characterization of the native OGDHc. α-Ketoglutarate, NAD^+^ and CoA were used at the concentrations shown in each plot accordingly, the velocity was normalized to 0-1 and a line is connecting each point, with different symbols annotating the data points for the three independent biological replicates, as annotated in the figure panel. **C** Graph displaying the change in reaction velocity (after normalization to 0-1) in relation to temperature for *C. thermophilum* as compared to a yeast equivalent sample, with different symbols annotating the data points for the three independent biological replicates, as annotated in the figure panel. The *K*_M_ values shown in plots **B** and **C** were obtained by the Burk-Lineweaver plots shown in Supplementary Fig. 2B and the gray background for each graph and the black bars at the bottom graph represent the standard deviation derived from *N* = 3 independent biological replicates and 2 technical duplicates for each replicate. All values shown here are listed in Supplementary Data 1.

The thermophilic fungus *C. thermophilum* was grown at 54 °C for 20 h before lysing and fractionating by size-exclusion chromatography (SEC) 8 g of cell fresh weight (Methods). *C. thermophilum* hyphae (Fig. 1C–G) are rich in mitochondria, as validated by confocal and electron microscopy methods (Fig. 1C–G). Mitochondria exhibit the expected lamellar, ribbon-like cristae, forming parallel stacks in cross section^17^ (Fig. 1F, G). A high abundance of mitochondria (Fig. 1F, G) is consistent with the increased respiratory rates of thermophiles^18^ and motivated us to derive a cell extract enriched in mitochondrial activities in which the functional OGDHc presents itself in high abundance^14^ with all its known subunits (E1o, E2o, E3)^19^. Here, we verified the presence of all enzymes via Western Blotting (WB) (Fig. 2A, Supplementary Fig. 2A) and proceeded with quantitative kinetic parameter characterization for all of the metabolon’s soluble substrates, namely NAD+, α-ketoglutarate (α-KG) and coenzyme-A (CoA). OGDHc catalyzes the conversion of α-ketoglutarate (α-KG) to succinyl-CoA through a multiple-step reaction involving different co-factors (Fig. 1B): thiamine diphosphate (ThDP) binds to the E1o, a lipoate covalently attached to the lipoyl-binding domain (LD) of the E2o, whereas FAD binds to its respective site at the E3. E1o and E2o are unique for this complex, but E3 is shared amongst all oxo-acid dehydrogenase complexes. In-fraction *K*_M_ values were determined at [149.80 ± 41.78] μΜ, [146.11 ± 46.15] μΜ and [22.81 ± 11.93] μΜ respectively (Fig. 2B, Supplementary Fig. 2B, Supplementary Data 1) and are comparable to those from other thermophilic counterparts^20,21^. To accurately display the advantage of an active cell extract derived from a thermophilic eukaryote, we repeated the characterization and performed a comparison of the reaction velocity for α-KG in a temperature gradient between a *C. thermophilum* and a *S. cerevisiae* equivalent extract (Fig. 2C, Supplementary Data 1). The derived data clearly displays an increase of reaction velocity for the *C. thermophilum*- derived cell extract as contrasted by the velocity decrease of the yeast equivalent sample showing the suitability for structural analysis of the cell extract derived from a thermophilic organism. To our knowledge, measurement of kinetic parameters for all substrates of OGDHc in a single endogenous specimen has not been previously reported. Our results set a benchmark for future functional comparisons of keto acid complexes. Our findings also demonstrate that a single cellular fraction is kinetically active, scalable due to its biochemical nature, and exploitable for product formation without requiring further purification or enrichment schemes. Importantly, such an active cell extract can be visualized by cryo-EM to deepen structural understanding into the active complex.

### The 3.35 Å cryo-EM structure of the native, endogenous eukaryotic OGDHc core from the cell extract elucidates structural features

**Fig. 3:**
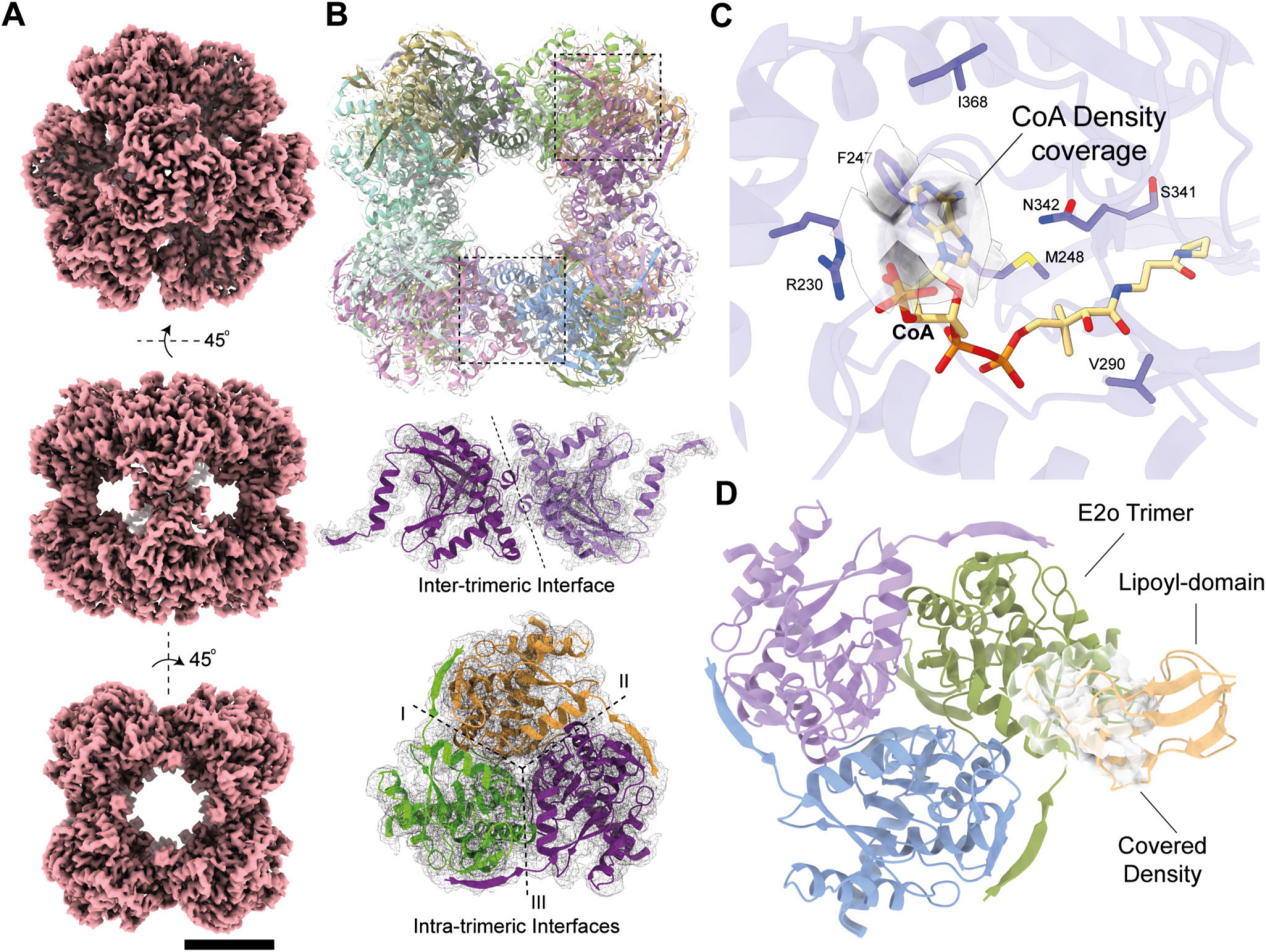
The Cryo-EM structure of the OGDHc E2o 24-mer core. **A** The 3.35 Å (0.143 FSC) cryo-EM map of the *C. thermophilum* OGDHc E2o core. Scale bar: 5 nm. **B** The reconstructed model of the *C. thermophilum* E2o core, fitted in the cryo-EM map. Beneath, the inter- and intra-trimeric interfaces can be observed in isolation. **C** The CoA binding region of the E2o, with the annotated amino-acid sidechains that participate in its coordination. Additionally, resolved density in the pocket may correspond to a bound CoA. **D** Resolved density in the periphery of the E2o vertex trimer, possibly belonging to a bound E2o lipoyl domain.

To structurally characterize the OGDHc metabolon, we performed an intensive cryo-EM data acquisition of the characterized active extract. The structure determined for the E2o core reached 3.35 Å resolution (Supplementary Fig. 3A, Supplementary Table 1) enabling de novo model-building and considerably improving the resolution over that of the previously reported E2o core^16^ (Supplementary Fig. 3B). The EM map (Fig. 3A) displays higher-order structural features, *e.g*., inter-, and intra- trimeric interfaces (Fig. 3B), captures secondary structure elements and accurately places amino-acid side chains (Supplementary Fig. 3C). We were able to identify the E2o’s catalytic active site and fully model side-chain conformations of the amino-acids participating in CoA binding which are conserved across oxo-acid dehydrogenases. F247 has an outward-facing orientation to accommodate the CoA (Fig. 3C), interacting and stabilizing the CoA 3’,5’-adenosine diphosphate group via π-stacking. The limited presence of electron density for the CoA pantoic acid, β-alanine, and cysteamine components underlies the inherent flexibility of endogenously bound coenzyme A with implications for function^22^.

Another density is observed proximal to the E2 lipoyl binding site, and in which, the lipoyl- domain (LD) of the E2o *N-ter* region can fit (Fig. 3D); This observation is in line with previously published data, where in the bacterial endogenous pyruvate dehydrogenase complex (PDHc), which also forms a cubic core – a feature of keto-acid complexes across kingdoms – an equivalent density was also attributed to the LD^23^. A bound LD was also structurally refined in the eukaryotic PDHc E2 active site in a similar, stable conformation^24^. In the eukaryotic, native, and active OGDHc presented here, this density persists, albeit to lower resolution, and the LD can be partially accommodated (Fig. 3D). A major difference lies in the fact that, compared to bacterial PDHc, which could be trapped in a proposed resting state with all LDs bound to the E2 active sites, the present eukaryotic OGDHc highlights an alternative resting state where LDs are sub-stoichiometrically and transiently interacting with the E2 core. Overall, this OGDHc core structure comprises the highest resolution reconstruction reported to date for a protein community member characterized in native cell extracts. Besides obvious high-resolution features, the higher-order interfaces and LD/CoA active sites are defined at high resolution, while, due to its endogenous nature, low-resolution densities appear for the flexibly bound CoA and the sub-stoichiometrically-bound LD.

### Revealing E2o dihydrolipoyl succinyltransferase core compaction in the context of the native metabolon

**Fig. 4:**
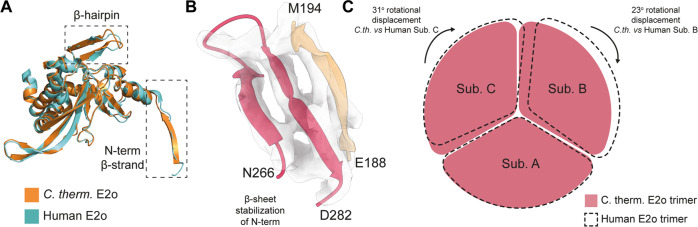
Structural adaptations of the *C. thermophilum* E2o in comparison to its mesophilic counterpart. **A** *C. thermophilum* E2o protein (orange) aligned with its human counterpart (cyan). Noticeable differences include a tighter turn conformation resulting in better alignment of the *C. thermophilum* β-hairpin model and the β-strand conformation of the *C. thermophilum N-ter*. **B** The β-hairpin motif of N266-D282, along with the *N-ter* β-strand E188-M194 form a β-sheet that contributes to the core’s overall stability. **C** Schematic representation of the human E2o vertex trimer rotational displacement in comparison to the experimentally resolved *C. thermophilum* E2o trimer, displaying a “loose” conformation for the mesophilic counterpart.

The OGDHc dihydrolipoyl succinyltransferase (E2o) reconstruction highlights broad similarity with the human overexpressed, inactive counterpart in terms of overall fold but with localized adaptations (Fig. 4). The E2o *N-ter* in previously published structures does not acquire a specific fold (Fig. 4A). By contrast, in *C. thermophilum* this element obtains a β-strand (E188-M194) conformation which, along with a β-hairpin motif of residues N266 - D282 creates an extended β-sheet (Fig. 4B). This adaptation contributes to the stabilization of the core via extensive hydrogen bonding and affects subunit inter-communication in the higher-order state of the 24-meric cubic E2o core. This fold imposes a condensation of the E2o core trimer when compared to its previously published human counterpart, translated in a lower centroid distance across subunits (Fig. 4C, Supplementary Fig. 4A). Subsequent energy-based scoring of the human^10^ and C*. thermophilum* inter- and intra- trimeric interfaces (Supplementary Fig. 4B, C, Methods) show consistently higher scores for the native, thermophilic E2o interfaces. Considering that buried surface area is proportional to the experimentally measured dissociation constants (*K*_D_) of biomolecular complexes that do not undergo large conformational changes^25^, our results suggest a stronger binding of *C. thermophilum* E2o enzymes. In line with this notion, *C. thermophilum* intra- and inter-subunit interfaces are 1.7 and 1.5 times larger than the corresponding interfaces of the human E2o core (Supplementary Fig. 4B, C) and include extensive charge-charge interactions (Supplementary Fig. 4B, C). Another consequence of the aforementioned compaction is the induced confinement of the E2o *N-ter* LD. An antiparallel β-sheet has greater stability as compared to the previously resolved loop conformation^26^. Antiparallel and mixed sheets can withstand both distortions (twisting and β-bulges) and exposure to solvent; therefore, the newly identified structural element can serve as an anchor point for restraining conformations explored by the flexible region connecting the two ordered E2o domains, *i.e*., the succinyltransferase forming the E2 core and the LDs carrying the intermediates.

### Electrostatic complementarity of comparable magnitude governs the 3D architecture of the OGDHc reaction interfaces

**Fig. 5:**
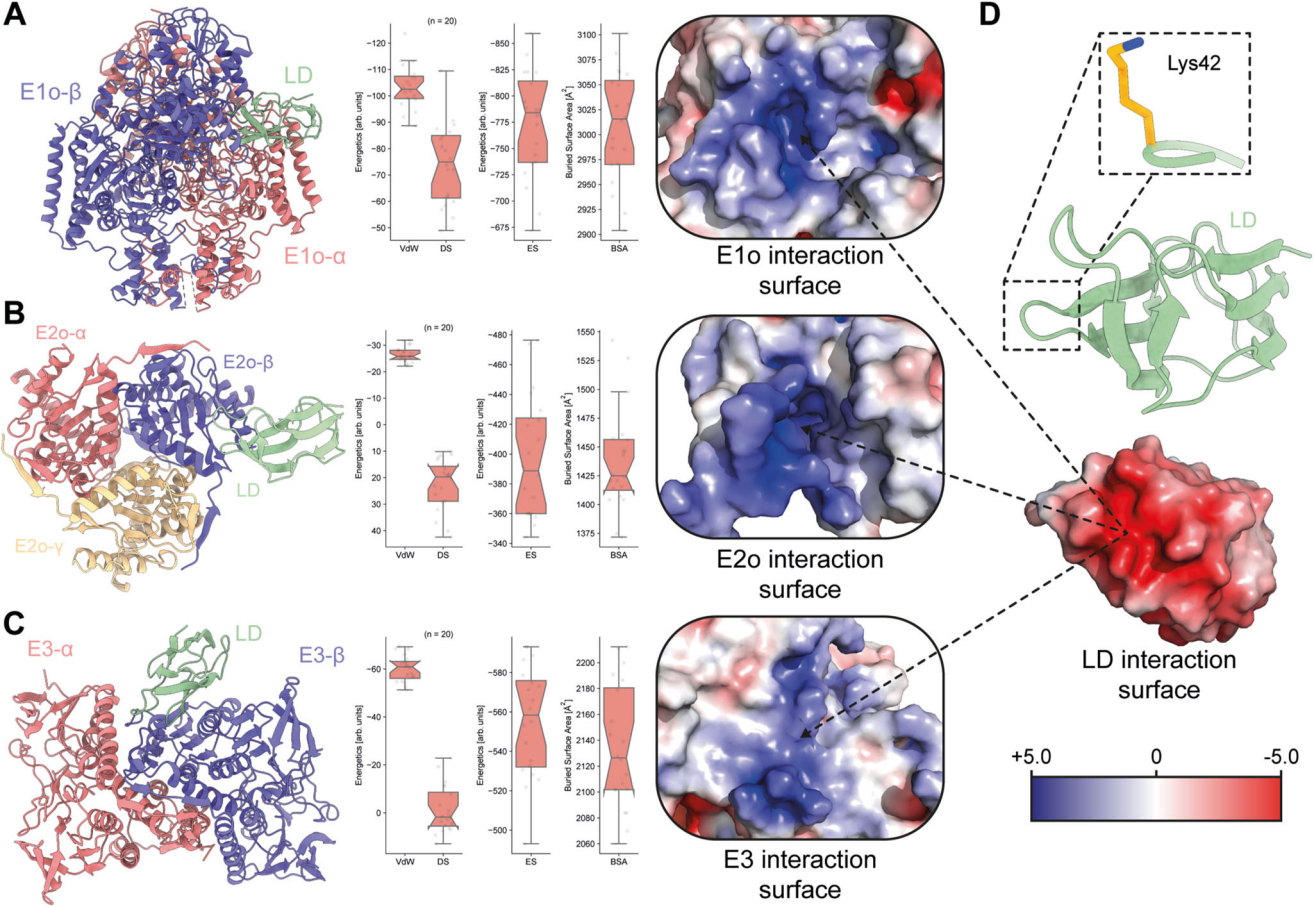
Electrostatic interactions at the interfaces formed by the OGDHc components. **A** AI-derived model of the E1o-LD interaction. Overall model, energetics (VdW Van der Waals interactions, DS Desolvation energy, ES Electrostatics) and buried surface area (BSA), and the overall charge of its interaction interface with the LD can be seen in left, middle and right respectively. **B** AI-derived model of the E2o-LD interaction. Overall model, energetics and buried surface area, and the overall charge of its interaction interface with the LD can be seen in left, middle and right respectively. **C** AI-derived model of the E3-LD interaction. Overall model, energetics and buried surface area, and the overall charge of its interaction interface with the LD can be seen in left, middle and right respectively. **D** AI-derived model of the ordered LD domain of E2o. Lys42 carries the lipoyl-moiety. A highly negatively charged interaction interface is observable. Original values for plotting can be found in Supplementary Data 5.

During catalysis (Fig. 1B) in the active OGDHc metabolon that we captured (Fig. 2B), the flexible E2o arm holding the LD shuttles the succinyl- intermediate from the E1o to the E2o active sites and is re-oxidized by the E3^27^; Therefore, the lipoylated lysine of the LD must have unhindered access to each enzymatic site. To structurally characterize these transient interfaces, AlphaFold-Multimer modeling^28^ of the three metabolon-embedded component interfaces (E1o-LD, E2o-LD and E3-LD) was performed. The models generated for all three complexes (Fig. 5A–D, Methods) are of high quality, and their predicted interfaces confidently rank within experimental error (Supplementary Fig. 5A, B). In addition, (a) the fit of the LD in the proximal density of the E2o core derived by cryo-EM (Fig. 3D) is similar to the one predicted by AI in terms of positioning (Fig. 5B) and (b) the distance of the lipoylated lysine to the cofactor or active site for all generated interfaces is in the range of 10–25 Å (Supplementary Fig. 6A–D). This range corresponds to the length of the lysine side chain and the lipoate and also reflects the intrinsic flexibility of the interacting proteins^15^, also observed in a recent bacterial PDHc E2o-LD interface^24^. E1o forms a homodimer (Fig. 5A), with two thiamine diphosphates (ThDPs), and potentially two LD binding sites localized at the E1o inter-chain interface (Fig. 5A). The lipoylated lysine distance to the ThDP C2 atom that is succinylated during α-ketoglutarate decarboxylation is biochemically feasible (*d* = 14 Å) (Supplementary Fig. 6A). E2o binds a single LD per monomer (Fig. 5B), and the lipoyl-lysine is proximal to the bound CoA, with a lysine Cα-CoA thiol group distance of *d* = 10 Å as well (Supplementary Fig. 6B). Lastly, the E3 active site locates a conserved disulfide bridge and a histidine in proximity involved in the E3-mediated LD-re-oxidation reaction^29–31^. The AI-based E3-LD complex shows that the lipoylated lysine is in reasonable distance to access disulfide bonding in the E3 active site (*d* = 23 Å) (Supplementary Fig. 6D). Note that a second, alternate conformation model for both E1o-LD and E3-LD complexes was generated (Supplementary Fig. 5B, Supplementary Fig. 6C, D) but did not satisfy the above-mentioned biochemical constraints essential for an active metabolon (Fig. 2B).

The biochemically- (E1o-LD, E2o-LD and E3-LD) and cryo-EM- (E2o-LD) validated AI-generated metabolon-embedded interfaces share a common characteristic, revealed after subjecting each to energy-based refinement (Fig. 5A–C): All interfaces are governed by strong electrostatic interactions of comparable magnitude (Fig. 5A–C) that comprise a signature energetic component for transient interfaces with high on/off rates^32^. Complementary electrostatics (Fig. 5D) is a collective characteristic in metabolons shuttling intermediates via a “swinging arm” mechanism^33^ and other metabolic processes requiring rapid on/off switching^34,35^. Indeed, electrostatic potential maps highlight extensive positively charged regions for the LD binders (Fig. 5D). These surfaces attract and accommodate the LD’s negatively charged surface and overall contribute to the structural compaction of oxo acid dehydrogenase complexes^36^ that is a key principle to achieve metabolic channeling via the “swinging arm” mechanism^37^.

### Mapping in-extract protein communities, validating AlphaFold2 models and identifying the OGDHc E3BP by proteomics and crosslinking mass spectrometry

To elucidate the broader proteome and its interactions within the active extract, we performed mass spectrometry-based crosslinking experiments directly in the extract (Methods) while benchmarking crosslinker concentrations (Supplementary Fig. 7A, B). Overall, with an FDR of 2% at the peptide recovery level (Methods), we retrieved 4949 residue-residue crosslinks (3632 intra- and 1317 inter- residue crosslinks), of which 99.4% mapped to *C. thermophilum* polypeptide chains (Supplementary Data 2). Out of a total of 2091 polypeptide chains, 505 polypeptide chains were crosslinked across 2 biological and 2 technical replicates (Supplementary Data 2). This shows that 29.1% of the total *C. thermophilum* proteome organizes in cellular communities^38^ which are significantly larger than previously described^19^.

**Fig. 6:**
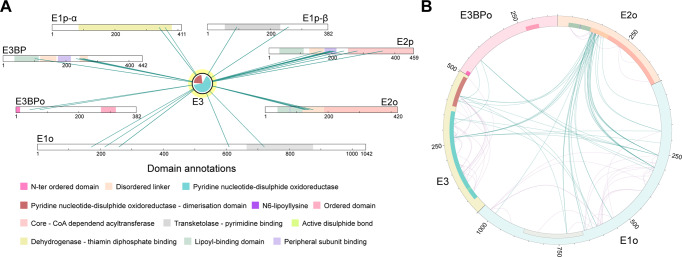
Crosslinking mass spectrometry (crosslinking-MS) of the native cell extract fraction uncovers inter-molecular interactions. **A** Inter-crosslinks of E3 reveal a plethora of interaction partners. **B** The OGDHc protein components are highly interconnected. Blue lines represent the inter-protein crosslinks and purple the intra-protein crosslinks.

Network analysis identified 54 cellular communities from diverse cellular compartments (Supplementary Data 2) in which 1488 identified members participate. Subunit coverage across communities is high (67% +/−24%) and includes examples such as the complete cytoplasmic (*N* = 128, 96% of subunits) and mitochondrial (*N* = 74, 99% of subunits) translation apparatus. Additionally, we identify all subunits of the pyruvate dehydrogenase/tricarboxylic acid cycle community crosslinked together (PDHc/TCA, *N* = 25, 100% of subunits, Supplementary Data 2). Within the PDHc/TCA community, crosslinks define interactions within and across eleven participating enzymes (122 intra- and 169 inter-links, Supplementary Data 2). The E3 protein displayed the largest number of inter-crosslinks identified in the experiment (*N* = 88) and was found in proximity to seven other community members (Fig. 6A), indicating its diverse role in mitochondrial metabolism; E3 proteins explore proximal spatial positions with all subunits of PDHc (E1p, E2p, E3BP), OGDHc (E1o, E2o), and a subunit of the hypothesized mitochondrial heme metabolon^39^, interacting within the latter with a putative holocytochrome c synthase (HCC) (Fig. 6A). However, sequence analysis highlighted a fused, misannotated ribosomal protein at the HCC *N-ter* (Supplementary Fig. 8) that corresponds to the elusive eukaryotic KGD4 OGHDc subunit^12,13^ that tethers the E3 to the OGDHc E2 core (residues M1 - T130). This *N-ter* shares high similarity with KGD4 from other eukaryotes (Supplementary Fig. 8). Reanalysis of MS data^19^ across cellular fractions shows strong co-elution of KGD4 with the rest of the OGDHc subunits in all three biological replicates (Supplementary Data 2). Because its function is essentially the same as the PDHc E3BP – tethering E3 to the E2 core – we designated the co-eluting protein as the *C. thermophilum* protein E3BPo.

### A crosslinking informed network elucidates interactions within the OGDHc metabolon

**Fig. 7:**
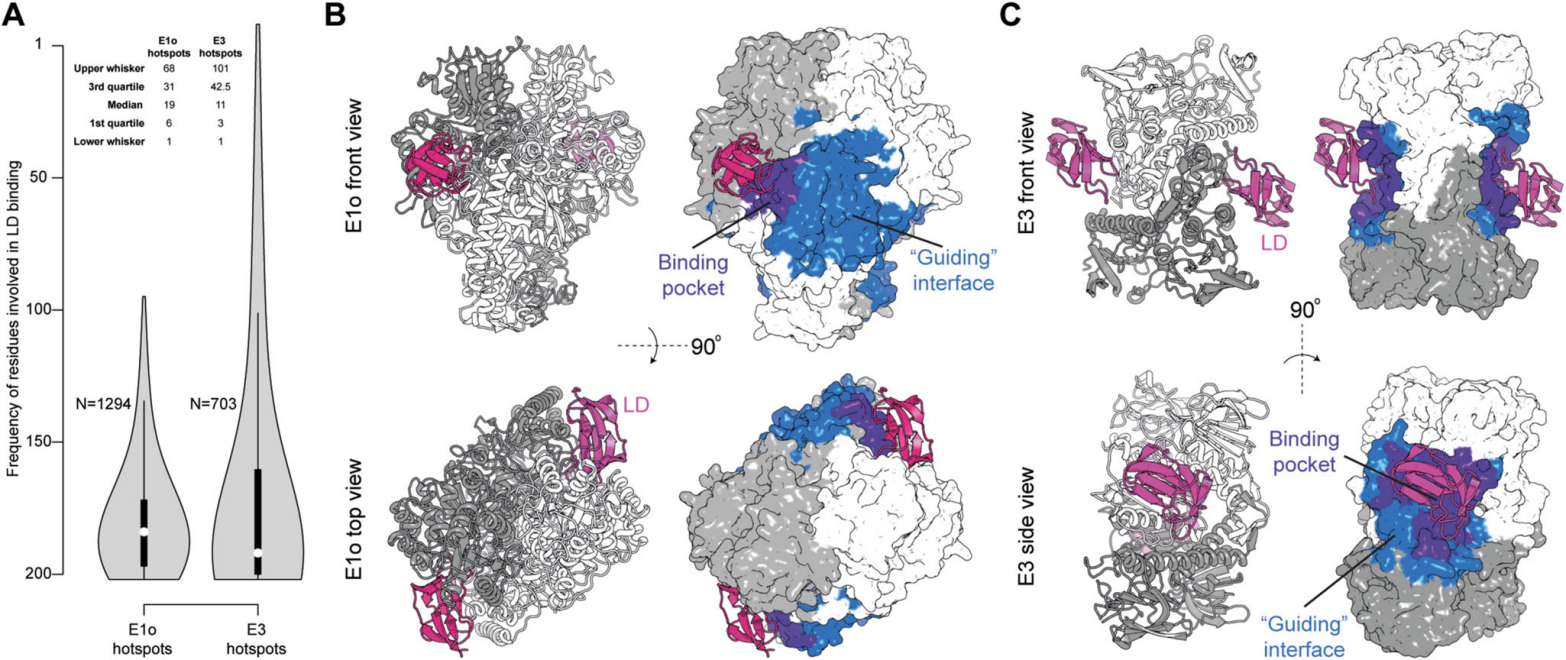
Crosslinking MS-driven docking reveals a “guiding” interface for LD binding. **A** Frequency plots of residues involved in the binding interface between the LD and E1o (upper) and LD and E3 (lower). **B** Residue frequencies of each residue involved in the interaction between the LD (pink) and the E1o (gray) reveal a “guiding” interface (blue) between the two that orients the LD towards its binding pocket (purple). **C** Residue frequencies of each residue involved in the interaction between the LD (pink) and the E3 (gray) reveal a “guiding” interface (blue) between the two that orients the LD towards its binding pocket (purple). Original values for plotting can be found in Supplementary Data 6.

44 inter- and 81 intra-molecular unique crosslinks were identified in the OGDHc components (E1o, E2o, E3, E3BPo) (Fig. 6B); intra-molecular crosslinks further corroborate the E1o, E2o and E3 molecular models (Supplementary Fig. 9A). In detail, 92,3%, 90% and 100% of mapped cross-links are satisfied for E1o, E2o, and E3, respectively. Distribution of cross-linking distances (Supplementary Fig. 9A, Supplementary Data 2) further recapitulate the expected log-normal distance distribution^40^ and corroborate the quality of the biochemically- and structurally-validated AI-derived models. Inter-molecular crosslinks overall define a relatively compact state of the OGDHc because enzymes not known to form physical interfaces (E1o/E3; E3BPo/E1o) are still present in relative proximity (Fig. 6B). The relative proximity of E1o and E3 to the E2o core point to the involvement of the *N-ter* flexible region of the E2 localizing downstream from the LD in the primary sequence (Fig. 6B). This result led us to utilize crosslinking-driven flexible molecular docking of the previously validated interaction models of the LD with the E1o and the E3 to elucidate interaction surfaces sampled by the LD (Fig. 7A–C).

We calculated the frequency of E1o or E3 residues involved in LD binding from the docking results (Fig. 7A) after adapting our docking protocols for incorporating crosslinks mapping to disordered regions proximal to ordered domains in protein-protein interactions (Methods,^25^). Mapping the top 25% of residues involved in LD binding derived from 400 explicit-solvent refined molecular models illustrates a distinct attraction surface for the LD to eventually bind to the respective active sites (Fig. 7B, C). Active site residues are identified as frequently contacted for both E1o (Fig. 7B) and E3 (Fig. 7C), but the attracting surface for the E1o is more diffuse in terms of recovered hot-spots as compared to the one calculated for the E3 (Fig. 7B, C). This higher signal diffusion for LD binding in the E1o suggests that the proximal surface may guide the LD towards the E1o binding pocket as it is rather deeply buried (Fig. 7B). For the E3, instead, the “guiding surface” is much more confined (Fig. 7C) at a narrow space equilaterally extending ~5 Å from the flat E3 active site. These results point to distinct LD attraction mechanisms for the peripheral E1o and E3 subunits governed by flexible regions positioned downstream from the LD.

Intra-crosslinks identified not only validate the presence of the participating proteins and their AI-derived structures (Supplementary Fig. 9A), but also confirm that they form multimeric structures; intra-crosslinks for E3BPo were not identified (Fig. 6B), and this may be an indication that the E3BPo takes part in the formation of the complex as a monomer; per E3BPo copy, a single E3 is tethered via its flexible *N-ter* region to the E2 core^13^. Translating iBAQ scores into qualitative stoichiometric data as previously done for PDHc^14^ points to an exact stoichiometry of 24 E2o subunits, validated by cryo-EM; and relative stoichiometries of <10 E1o dimers, and ~4 E3BPo, tethering 4 E3 dimers in total (Supplementary Fig. 9B, C). Because E3 is also in proximity to the E2o disordered regions and its proximal E2o LD domain, it is well possible that more E3s can be accommodated. However, considering that the E2 core may recruit up to 48 molecules, totaling a metabolon of 96 polypeptide chains (24 E2o and maximum 24 E3BPo monomers, each tethering 12 E1o and 12 E3 dimers, respectively), relative stoichiometries reveal substoichiometric composition of the peripheral OGDHc subunits. This points to the presence of unbound flexible *N-ter* E2o core regions involved in LD trafficking.

### Cryo-EM and computational analysis of E1o, E2 and E3 proximity in the context of the OGDHc metabolon

**Fig. 8:**
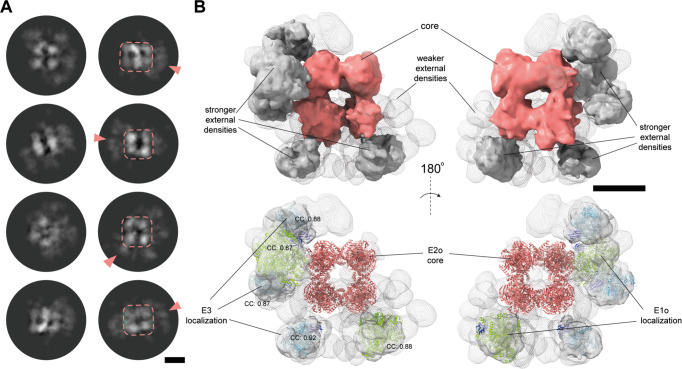
The asymmetric reconstruction of the complete OGDHc allows for the localization of its external subunits. **A** 2D class averages of the OGDHc present a core of high signal, surrounded by more diffused but still well-defined signal on the periphery. Scale bar: 5 nm. **B** The asymmetric 3D reconstruction of the OGDHc displays a strong density corresponding to the E2o core, and a mix of weaker and stronger external densities. In the stronger densities, E1o and E3 dimers can be localized with high confidence. Scale bar: 10 nm.

To elucidate the higher-order architecture of the OGDHc within the extract, we performed asymmetric reconstructions from the single-particle data, leading to 2D projections where external densities were visible (Fig. 8A). In these external densities, the identified E1o and E3 dimers should be located, each tethered via either the flexible regions of the *N-ter* of the E2o (Fig. 6B) or, in the case of E3, the flexible E3BPo (Fig. 6B,^13^). The cryo-EM map (Fig. 8B) was determined at 21 Å resolution (Supplementary Fig. 3B, Supplementary Table 1), exhibiting densities around the E2o core, in a clustered and not in a diffused fashion (Fig. 8B). Increasing density thresholds of the cryo-EM map showed additional, weaker densities (Fig. 8B). Systematic fitting of AlphaFold2-derived E1o-LD and E3-LD models of *C. thermophilum* (Fig. 8B, Supplementary Fig. 10A, B, Supplementary Data 3) in the cryo-EM map resolved those molecules’ localization and suggests they are present in the periphery and not on or within the core densities. In addition, persistent docking solutions that recapitulated the positions of E1o and E3 in the map were calculated from the systematically fitted structural models associated with high cross-correlation values (Supplementary Data 3, Supplementary Fig. 10A, B): Overall, 2 E1o and 3 E3 dimers could be resolved within the calculated external densities.

**Fig. 9:**
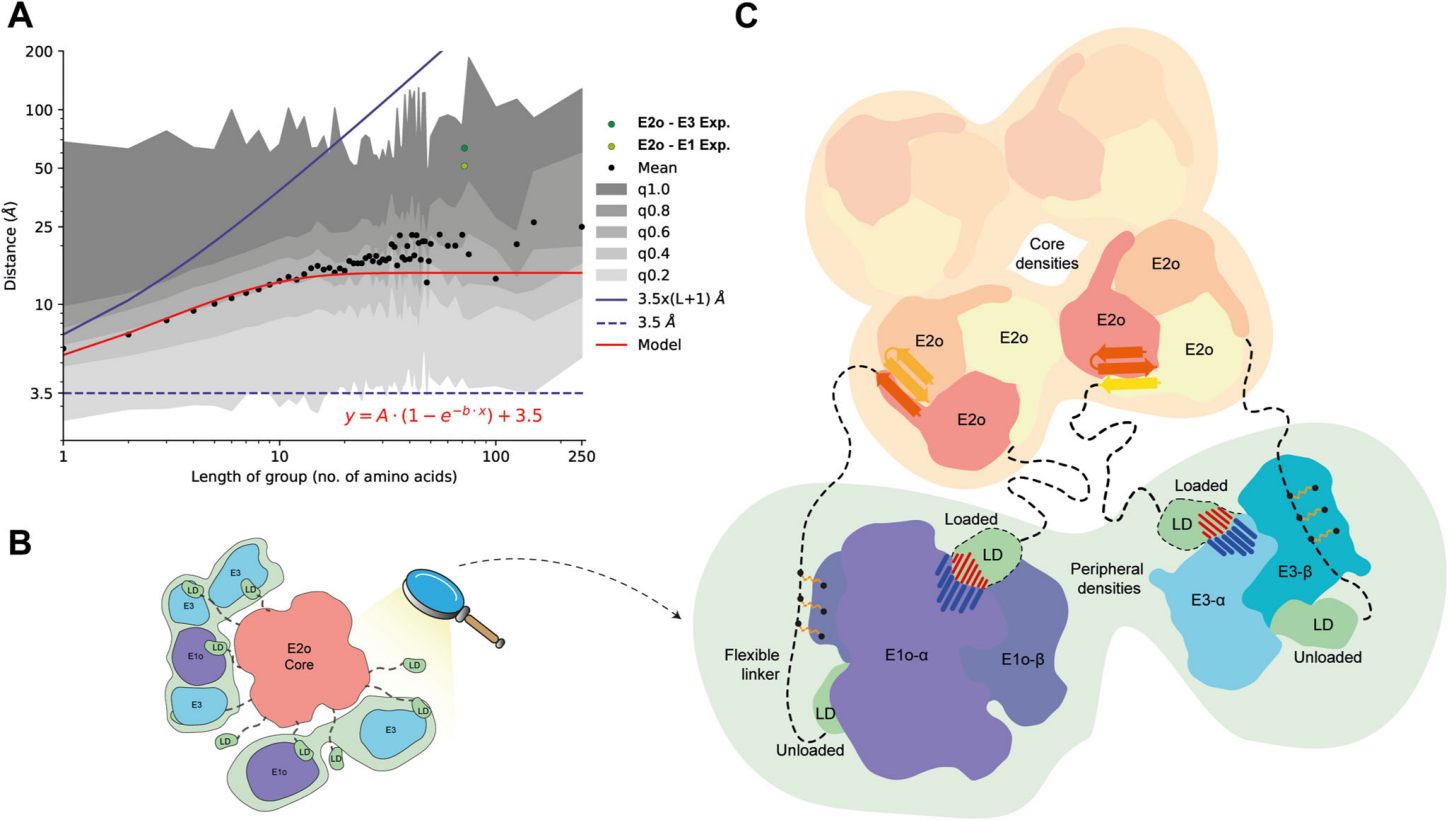
Flexible linker distances and a comprehensive model for the organization of the OGDHc. **A** Graph representing the mean distance values of the binned groups of all unresolved amino-acid sequences belonging to all protein structures deposited in the PDB. Black dots represent the mean value of a length of amino-acids group, with varying scales of gray the standard deviation after the integration of each quartile of total data, as denoted in the plot legend. The red line represents a fitted model that describes the relationship between the distance and the length of amino-acid groups. With light green the experimentally measured average distance between the *N-ter* of the resolved core domain of the E2o and the *C-ter* of the LD that is bound to a fitted E1o dimer on the periphery, while dark green represents the same average distance experimentally measured between the *N-ter* of the resolved core domain of the E2o and the *C-ter* of the LD that is bound to a fitted E3 dimer. The blue line represents the theoretical distance of an amino-acid sequence, while the dashed blue line represents the theoretical lower limit of any amino-acid sequence. **B** Schematic representation of the peripheral subunit organization of the resolved OGDHc. **C** Model describing all the novel observations concerning the organization of the OGDHc. The *N-ter* β-sheet conformation of the E2o core vertex trimer helps stabilize and compact the 24-mer E2o core, while simultaneously orienting the flexible linker that connects the LD domain. Cross-linking data reveals a fairly stable interaction between the flexible linker and the peripheral subunits, possibly hinting at a structural role of an un-loaded LD domain, while another, loaded LD domain performs the reaction cycling. The interaction between the LD and the peripheral subunits is governed by strong electrostatic forces.

While the tethered E1o and E3 enzymes are asymmetrically distributed in proximity to the E2o core, they retain approximately equal distances from the core itself (Fig. 8B). The inherent flexibility of the tethering region in OGDHc does not reconcile with physical-chemical or statistical calculations for flexible protein regions of various categories (e.g., IDPs, molten globule, fully extended; Supplementary Fig. 10C). According to the E2o linker length measured in our derived 3D reconstruction (Fig. 9A, Supplementary Fig. 10C), OGDHc flexible regions have properties in-between calculated lengths of “stretched” IDPs (IDPs as defined in Marsh and Forman-Kay^41^) and “constricted” linkers (as defined by George and Heringa^42^). To elucidate whether distances measured between ordered OGDHc participating enzymes that are mediated by flexible regions can be recapitulated in the available structural data, we calculated the distance of amino acid residues that are resolved but are sequence-wise further apart due to an unresolved stretch in the structural data. While various technical reasons may account for the presence of this unresolved stretch, it may also be an indicator of structural disorder^43^. By analyzing all Protein Data Bank (PDB)^44^ structural data as of June 2022 (191,144 structures), results show dramatically confined distances between the ordered residues preceding and following the “missing” stretch (Fig. 9A). This means that current structural data in the PDB very rarely include interactions of the kind reported in our results (Fig. 8B, Fig. 9A). A relative “flattening” of calculated distances for absent polypeptide stretches of more than 25 amino acid residues is evident (Fig. 9A), and maximum Ca-Ca distances reported average at ~30 Å, which are substantially shorter than those in the OGDHc metabolon.

## Discussion

Our combined findings expand our understanding of the proposed architecture of the oxoglutarate dehydrogenase complex in the context of a succinyl-CoA-producing cell extract (Fig. 9B, C). The complex’s core is a robust cubic ultrastructure where the E2o trimers that comprise the vertices are densely packed with the help of multi-subunit secondary structure elements (core domain *N-ter* β-sheet). This dense core packing could either be a thermophilic adaptation, as previously shown in the case of the related pyruvate dehydrogenase complex^14,15^ or a general feature of the endogenous OGDHc. However, although OGDHc is considered similar to PDHc, the endogenous E2 *N-ter* conformations are distinct: in OGDHc, the E2 *N-ter* is folded in a β-strand conformation, part of an inter-chain β-sheet; in endogenous PDHc, this *N-ter* element is flexible. Such structural adaptations expand our view on possible specificity determinants for the binding of distinctly loaded LDs to different E2 cores in oxo acid dehydrogenases.

Another observation we derived is that the same structural element confines the available space of the extended *N-ter* flexible linkers that tether the LD domain which is the crucial domain for the transport of the substrates across sequential active sites that take part in the reaction, at around 50–100 Å for the LD-E1o interaction and 60–70 Å for the LD-E3 interaction. It is also of note that electrostatic complementarity drives the binding of the LD to its various binders (E1o, E2o, E3). The flexible linker and LD of E2o, based on our XL-MS data, may also act as an “anchor” that, in the absence of specific peripheral subunit binding domains in the E2o *N-ter* sequence, maintains the E1o and E3 subunits in proximity to the E2o core. The disordered *N-ter* domain of E1o was recently resolved to interact with the E1o-LD binding site^45^ and may further aid in organizing E1o in proximity to the E2o core by interacting with other LD-binding interfaces in the metabolon. Both E1o and E3, being homodimers, contain dual LD-binding sites suggesting one playing an active role in the reaction, while the other can maintain a highly-charged complementary electrostatic tethering with a proximal “unloaded” LD domain. This “unloaded” LD domain may participate as an “anchor”, possibly also assisted by other weaker interactions of the flexible linker with surface residues of the peripheral subunits as well as the identified E3BPo protein, also captured in our XL-MS data to organize the E3 protein. The “structural” role of the extra LD domains can also be hinted by the sub-stoichiometric relationship between the core E2o and the peripheral E1o-E3-E3BPo subunits as quantified by the MS data presented here. Combining molecular biology methods to overexpress E1o, E3, E3BPo subunits and perform mixture experiments with the OGDHc-containing cell extract fraction would allow to possibly gain deeper insights into the interplay of stoichiometry, interactions and adaptations of the molecular structure of the metabolon. This work, aided by quantitative proteomics with labeled peptides to accurately determine in-extract stoichiometries, and therefore, concentrations, would provide important material to understand OGDHc function with cryo-EM.

Overall, in this work, we characterized the complete contents of a cell extract with succinyl-CoA producing capabilities, and elucidated the biochemical function and novel structural features of the reaction’s main player, the OGDHc and its components, E1o, E2o, E3, E3BPo. Our work gives rise to questions that need to be addressed: Is there in-fraction interplay among the multiple protein communities present? How can their biochemical and structural relationships be characterized? These questions can only be answered by employing automated, high-throughput cryo-EM of a fully, high-density, crosslinked cell extract, combined with more large-scale data collection to tackle the issues of complex flexibility and relative low abundance of individual elements. Advances in instrumentation^46^, data collection^47^ and analysis^48,49^, as well as the advent of precise AI-driven modeling^50^, will allow for the streamlining of the characterization of native cell extracts. It is possible that in the near future our approach will lead to new paradigms of enzymatic analysis in the context of other cellular metabolons that remain elusive.

## Methods

### Model organism culture

*Chaetomium thermophilum var. thermophilum* La Touche 1950 (*Thermochaetoides thermophila*^51^) was acquired from DSMZ (Leibniz Institute DSMZ-German Collection of Microorganisms and Cell Cultures, Germany), and the preserved in freeze-dried ampoule spores were cultivated as directed by company guidelines (DSMZ Media list Medium 188, temperature: 45 °C). After initial cultivation, the mycelium was propagated in liquid Complete Culture Media (CCM), containing per 1,000 mL of ddH_2_O: 5.00 g tryptone, 1.00 g peptone, 1.00 g yeast extract, 15.00 g dextrin, 3.00 g sucrose, 0.50 g MgSO_4_ × 7 H_2_O, 0.50 g NaCl, 0.65 g K_2_HPO_4_ × 3 H_2_O and 0.01 g Fe_2_(SO_4_)^3^ × H_2_O. CCM final pH was adjusted to 7.1. Final cultures were performed as follows: Solid media plate cultures: liquid CCM was supplemented with 15.00 g of Agar/1000 mL ddH_2_O and the plates were then inoculated with mycelium and grown at 54 °C. Liquid media cultures: 2000 mL Erlenmeyer flasks were filled to 40% of total volume (800 mL) with liquid CCM media, small pieces of freshly grown mycelium from Agar plates were added and then incubated under shaking at 110 rpm and 10% CO_2_ for 20 h.

### Cell imaging

Fluorescence microscopy imaging was performed as follows: A Zeiss LSM880 (Carl Zeiss, Germany) with a 40X objective lens without immersion (plan-Apochromat 40X/0.95 N.A.) was used to image the samples and images were acquired with the ZEN Black image analysis software (Carl Zeiss, Germany). For membrane staining with FM 4-64 (ThermoFisher Scientific, USA), the dye was diluted in DMSO to a stock concentration of 10 mM; 1 μL of stock solution was then added to 1 mL of *C. thermophilum* liquid cell culture, reaching a working concentration of 10 μM. Samples were imaged after 2–3 m of incubation time. For mitochondria staining with MitoTracker orange (ThermoFisher Scientific, USA) the dye was diluted in DMSO to a stock concentration of 10 μM; 5 μL of stock solution were then added to 1 ml of *C. thermophilum* liquid cell culture, reaching a working concentration of 50 nM. Samples were imaged after 10 m of incubation time. Transmission electron microscopy (TEM) imaging was performed as follows: freshly propagated liquid culture *C. thermophilum* filaments were fixed with 3% glutaraldehyde (Sigma, Taufkirchen, Germany) in 0.1 M sodium cacodylate buffer (SCP; pH 7.2) for five hours at room temperature. After fixation the samples were rinsed in SCP and postfixed with 1% osmiumtetroxide (Roth, Karlsruhe, Germany) in SCP for one hour at room temperature. Subsequently the samples were rinsed with water, dehydrated in a graded ethanol series, infiltrated with epoxy resin according to Spurr (1969)^52^, polymerized at 70 °C for 24 h and then cut to 70 nm ultra-thin sections with an Ultracut S ultramicrotome (Leica, Germany). After cutting, the sections were applied on copper grids with formvar coating and uranyl acetate and lead citrate was added for post-staining in a specialized EM-Stain device (Leica, Germany). For imaging, a Zeiss EM 900 TEM (Carl Zeiss, Germany) operating at 80 keV was used. All image processing was performed using the Fiji software^53^.

### Cell extract preparation

To prepare the cell extract, carefully grown mycelium^15^ was isolated with the use of a 180-μm-pore size sieve, then washed 3 times with PBS at 3000 × *g*, 4 min and 4 °C. After removing any residual moisture, the pellet was freeze-ground using a liquid N_2_ pre-chilled mortar and stored for later usage at −80 °C. Approximately 8 g of the freeze-ground material were lysed in 20 mL of Lysis Buffer (100 mM HEPES pH 7.4, 5 mM KCl, 95 mM NaCl, 1 mM MgCl_2_, 1 mM DTT, 5% Glycerol, 0.5 mM EDTA, Pefabloc 2.5 mM, Bestatin 130 μM, 10 μg mL^–1^ DNAse, E-64 40 μM, Aprotinin 0.5 μM, Pepstatin A 60 μM, Leupeptin 1 μM), with 3 repeats of 6.5 mps shaking speed for 25 s, 4 °C in a Fastprep cell homogenizer and 3 min of rest in ice after each repeat. A 4000 × *g* centrifugation step was used to pellet the larger cell debris and a 100,000 × *g* high-speed centrifugation was then performed. The resulting supernatant was filtered through a 100 KDa cutoff centrifugal filter and concentrated to 30 mg·mL^−1^.

The filtered, concentrated to 30 mg·mL^−1^ supernatant, was applied to a Biosep SEC-S4000 size exclusion column that is mounted to an ÄKTA Pure 25 M FPLC (Cytiva, USA) system via a 500 μL loop. The column was equilibrated with a filtered, degassed buffer containing 200 mM of CH_3_COO^−^NH4^+^ at pH 7.4 prior to sample application. Fraction volume was set to 250 μL and flow rate to 0.15 mL·min^−1^. Based on acquired MS data (see details below), fraction 6 was selected to be tested for suitability as a succinyl-CoA producing cell extract.

In order to produce an equivalent yeast sample for enzymatic activity comparison, the *Saccharomyces cerevisiae* strain from ATCC (American Type Culture Collection PO Box 1549 Manassas, VA 20108 USA; ATCC^®^ 24657TM) was cultivated in YPDG medium at 30 °C for 5 h, to a OD_595_ of 2.5 (early exponential phase), then harvested at 3000 × *g* for 5 min at 4 °C and washed with distilled water (resulting pellet ~7 g). The subsequent protocol until final sample preparation is identical to the *C. thermophilum* preparation described above.

### Oxoglutarate dehydrogenase (OGDHc) activity assays

The OGDH activity assay was adapted from^54^. The enzyme assay was prepared in a reaction volume of 100 µL at 4 °C, containing 100 mM NaCl, 30 mM K_2_HPO_4_ (pH 7.5), 2 mM MgCl_2_, 2 mM ThDP, 4 mM α-ketoglutarate, 3 mM NAD+, 0.4 mM CoA, and 4 µL Cell Counting Kit 8 and 2 µL cell lysate containing OGDH. For KM calculations, α-ketoglutarate was titrated from 50 to 5000 µM, NAD+ from 25 to 5000 µM, and CoA from 5 to 1000 µM. The reaction mixture (without α-ketoglutarate) was pre-incubated for 5 min at 37 °C for the substrate *K*_M_ calculations and at 25 °C to 65 °C with 5 °C intervals for temperature-dependent kinetic characterization, and the reaction started by the addition of α-ketoglutarate. Formazan product formation by WST-8 of the Cell counting kit 8 was monitored every minute for 1 h at 460 nm, and concentration calculated with the Lambert–Beer-Equation and the molar absorption coefficient of WST-8^55^ (ε = 3.07 × 104 M^−1^ cm^−1^). Due to substrate excess inhibition, reaction rates were plotted against substrate concentrations using the double reciprocal Lineweaver-Burk plot^56^, and *K*_M_ values were determined at the abscissa intersection point of the asymptotic linear regression.

### Immunoblotting experiments

For Western Blotting (WB) experiments, in-house casted, freshly prepared, 1 mm thickness gels were used with the following composition: separating phase: 370 mM Tris-HCl pH 8.8, 10% w/v acrylamide (37.5:1), 0.04% w/v APS, 0.002% v/v TEMED, 0.1% w/v sodium dodecyl sulfate (SDS) in ddH_2_O, stacking phase: 125 mM Tris-HCl pH 6.8, 5% w/v acrylamide (37.5:1), 0.04% w/v APS, 0.002% v/v TEMED, 0.1% w/v SDS in ddH_2_O. Samples were previously mixed with a 4x loading dye (250 mM Tris-HCl pH 6.8, 40% v/v glycerol, 20% v/v β-mercaptoethanol, 0.2% w/v bromophenol blue, 8% w/v SDS) and then incubated at 100 °C for 5 min. For each native sample, around 400 ng of protein was loaded in each lane and 5 μL of Precision Plus Protein™ All Blue Prestained Protein Standards (Biorad, USA) was loaded in each gel as a marker. Concentration of recombinant control samples was around 8 ng. After loading, gel electrophoresis followed in a 1X electrophoresis buffer, freshly diluted from a 10X stock (144 g Glycine, 30.3 g Tris-base in ddH_2_O) with an applied electrical field of 100 V for 1,5 h. A Trans-Blot^®^ Turbo Transfer System (Biorad, USA) was used to transfer the gel contents to a nitrocellulose membrane for 20 min, with a pre-set, 25 V (1 A) applied field. Blocking was performed for 1 h, under constant stirring in 5% w/v TBST/milk solution and then the membranes were incubated at 4 °C for 16 h with the primary antibody (all antibody concentrations applied were adjusted to 0.2 μg·mL^−1^, 2% w/v TBST/milk). The primary antibody was removed and three washing steps of 2% w/v TBST/milk followed. The membrane was then incubated with the secondary antibody (Goat Anti-Rabbit IgG, Abcam, ab205718, 0.1 μg·mL^−1^, 2% w/v TBST/milk) for 1 h. Three more washing steps of 2% w/v TBST/milk were applied and finally the membranes were screened with a ChemiDoc MP Imaging system (Biorad, USA) and freshly mixed ECL fluorescent mixture and optimal exposure times. All primary antibodies were tailor-made by GenScript (New Jersey, USA) and the antigen sequences used to generate the antibodies are described here: (a) E1o α/β protein has recombinant sequence that spans Met-E1o_611-818_-His6 and a molecular weight (MW) of 24,014.67 Da; (b) E2o protein has recombinant sequence that spans Met-E2o_39-420_-His6 and MW of 42,485.91 Da; and (c) E3 α/β protein has recombinant sequence that spans Met-E3_35-504_-His6 and MW of 51,341.91 Da.

### Cryo-EM sample preparation and data collection

For structural characterization of the succinyl-CoA producing cell extract, a 3.5 μL sample of final protein concentration of 0.3 mg·mL^−1^ was applied on a carbon-coated, holey support film type R2/1 on 200 mesh copper grid (Quantifoil, Germany) that was previously glow discharged under the following conditions: 15 mA, grid negative, 0.4 mbar and 25 s glowing time with a PELCO easiGlow (TED PELLA, USA). The grid was then plunge-frozen with a Vitrobot^®^ Mark IV System (ThermoFisher Scientific, USA) after blotting with Vitrobot^®^ Filter Paper (Grade 595 ash-free filter paper ø55/20 mm). In the chamber, conditions were stabilized at 4 °C and 95% humidity, while blotting parameters were set at 0 blot force and 6 s of blotting time. The vitrified grid was clipped and loaded on a Glacios 200 keV Cryo-transmission electron microscope (ThermoFisher Scientific, USA) under cryo and low humidity conditions. Images were acquired with the Falcon 3EC direct electron detector and the EPU software (ThermoFisher Scientific, USA) in linear mode and total electron dose of 30 e^−^/Å^2^. Before acquisition, the beam was aligned to be parallel and perpendicular to the sample, with a 2.5 μm diameter, while a 100 μm objective aperture restricted the objective angle. Complete acquisition parameters are listed in Supplementary Table 1.

### Image processing

All steps of image processing were performed with the cryoSPARC high-performance computing software version 3.3.1^57^. A dataset of 25,803 movies was imported to a dedicated workspace. The in-software patch motion correction (multi) and patch CTF estimation (multi) algorithms were employed to correct for beam induced motion and calculate for the CTF parameters of the micrographs respectively. After dataset curation, 24,300 micrographs were selected for subsequent steps of the image analysis process. Templates were created from EMD-13844^16^ with the create templates job (20 equally-spaced generated templates) and were employed for a picking job with the template picker, resulting in an initial dataset of 3,596,302 particles that was extracted with a box size of 208 pix. and then reference-free 2D classified in 200 classes. From the initial 2D classification, 71,912 particles were selected from 4 classes and subjected to heterogeneous refinement, further refining the final particle set to 52,034 particles after discarding particles that resulted in mal-formed OGDHc E2o core structures. The final particle set was then symmetry expanded with octahedral (O) symmetry and employed for the final core reconstruction with the local refinement (new) job, resulting in a OGDHc E2o core map of 3.35 Å resolution (Gold-standard FSC criterion 0.143) (Supplementary Fig. 3A). For the reconstruction of the complete OGDH complex, the 71,912 particles that were included in the core reconstruction were re-extracted with a larger box size of 288 pix in order to include signal for the subunits that are located in the periphery of the core. They were then re-classified in 20 2D classes and the classes that showed most prominent peripheral densities were used again as template for a new round of template picking, resulting in an initial particle set of 2,891,518 single particles. This particle set underwent 3 more rounds of 2D classification, always selecting towards class averages that displayed a robust core signal, but in addition to the core also displayed peripheral subunit signal, ending up with a set 52,551 particles that were finally used for a 3D classification job with 10 classes. Class 0, containing 5178 particles and displaying the most well-resolved peripheral densities, was finally used for homogeneous refinement, resulting in a OGDHc map of 21.04 Å resolution (Gold-standard FSC criterion 0.143) (Supplementary Fig. 9D) which was then utilized for all structural analysis. All map visualization was performed with the ChimeraX^58^ software package.

### Atomic model building and refinement

For refinement of the E2o core structure, the initial model (PDB ID: 7Q5Q) was fitted into the cryo-EM density using ChimeraX and then refined using iterative manual refinement with Coot^59^ and real-space refinement with Phenix^60^ with standard parameters. Visible density that could be mapped was extended towards the *N-ter* of the E2o, from M195 to E188.

### AI-based model generation and electrostatic surface calculation

A local installation of AlphaFold-Multimer^28^ was utilized in order to perform predictions of the E2o core vertex trimer that was modeled in the experimentally derived map, the E1o dimer (Uniprot ID: G0RZ09) and E3 dimer (Uniprot ID: G0SB20) in complex with the Uniprot-annotated E2o LD domain (residue numbers 40–115, Uniprot ID: G0SAX9). All AlphaFold2-based quality metrics (predicted aligned error (PAE) and predicted local distance difference test (plDDT) can be found in Supplementary Fig. 5A, B. In order to calculate and visualize all electrostatic surfaces, the APBS electrostatics plugin^61^ was used in PyMol (Schrödinger, USA).

### Energetic calculations and macromolecular docking

Two distinct protocols were applied for reported HADDOCK2.2 calculations: (a) HADDOCK refinement^62^. Here, the refinement protocol was applied to calculate and compare the energetics of interfaces between OGDHc components and its human homolog as well as the AlphaFold2-generated interfaces formed by the LD. In this refinement procedure, only the water refinement stage of the HADDOCK protocol was performed that showed to qualitatively correlate calculated energetics with binding affinities for transient protein-protein interactions^25^ skipping the docking step. For this, complexes were solvated in an 8 Å shell of TIP3P water. The protocol consisted of the following steps: (1) 40 EM steps with the protein fixed (Powell minimizer) and (2) 2 ×40 EM steps with harmonic position restraints on the protein (*k* = 20 kcal·mol^−1^ Å^−2^). For the final water refinement, a gentle simulated annealing protocol using molecular dynamics in Cartesian space is introduced after step (2). It consists of: (1) Heating period: 500 MD steps at 100, 200 and 300 K. Position restraints (*k* = 5 kcal·mol^−1^ Å^−2^) are applied on the protein except for the side-chains at the interface. (2) Sampling stage: 1250 MD steps. Weak (*k* = 1 kcal·mol^−1^ Å^−2^) position restraints are applied on the protein except for the backbone and side-chains at the interface; and (3) Cooling stage: 500 MD steps at 300, 200 and 100 K. Weak (*k* = 1 kcal·mol^−1^ Å^−2^) position restraints are applied on the protein backbone only except at the interface. A time step of 2 fs is used for the integration of the equation of motions and the temperature is maintained constant by weak coupling to a reference temperature bath using the Berendsen thermostat^63^. The calculations were performed with CNS^64^. Non-bonded interactions were calculated with the OPLS force field^65^ using a cutoff of 8.5 Å. The electrostatic potential (E_elec_) was calculated by using a shift function while a switching function (between 6.5 and 8.5 Å) was used to define the Van der Waals potential (E_vdw_). The structure calculations were performed on the HADDOCK web server at https://alcazar.science.uu.nl/ using the refinement interface. A total of 200 structures was generated for each complex. (b) HADDOCK flexible docking. The HADDOCK docking server was used, utilizing the guru interface. Here, distance restraints were used by applying the derived crosslinking data matching LYS residues of the LD and the E1, and E3, respectively. The distance restraints applied are included in the haddockparam.web files provided with this article. For the docking calculations, the guru interface was utilized by default, but search and scoring space were significantly expanded. This meant that it0 generated structures were increased to *N* = 10,000 and scored structures were increased to *N* = 400. Calculations of frequent amino acid residues involved in the binding of the LD were produced from the formed interfaces from the N = 400 final, water-refined docking solutions. An interface residue is considered if it is in proximity of 5 Å from any other residue from the LD. Highly frequent residues reported are the ones that belong to the top quartile (above 25%) in the calculated frequencies per residue and these were plotted with the BoxPlotR online webserver^66^.

### Peripheral subunit fitting

The OGDHc complex map that was generated from the cryo-EM experimental data was used to identify the placement of the peripheral E1o and E3 subunits, in complex with the E2o LD domain that were generated through AlphaFold-Multimer predictions as follows: the map was displayed in ChimeraX and after fitting the E2o core in the center, it was segmented into a “core” region and an “external” density region with the segment map tool. Then a fit search of 100 fits was performed for both E1o-LD and E3-LD complexes and cross-correlation (CC) values for each fit were listed and separated as fitting in the core or external density region. For statistical significance, the two CC fit groups were tested with single-factor analysis of variance (ANOVA) with the Analysis ToolPak in Microsoft Excel (Microsoft Corporation, USA) and values for the fits can be found in Supplementary Fig. 10A, B and Supplementary Data 3. CC plots were plotted with the BoxPlotR online webserver.

### Linker distance and rotational displacement calculations

*C. thermophilum* E2o linker distance calculations were performed as follows: (a) For the experimentally resolved distance measurements, after all peripheral subunits with bound LD were fit in the OGDH complex map, distance was measured with the “distance” command in ChimeraX, starting from the last resolved *N-ter* residue for each of the 3 E2o subunits of the visually inspected closest E2o core vertex trimer, to the first *C-ter* residues of the modeled LD domain bound to either E1o or E3 dimers on the periphery. All values were then averaged, and individual measurements and standard deviations calculated can be found in Supplementary Data 3. Theoretical calculations based on disordered linker length (73 a.a., Uniprot ID: G0SAX9) were then based on derived equations published by Marsh and Forman-Kay^41^, Wilkins et al.^67^ and George and Heringa^42^. Additionally, to further derive insights on disordered linker length based on experimentally resolved structures from the PDB, the complete PDB database, as of 1st Jun. 2022, was downloaded and a total of 191,144 mmCIF format files, containing more than half a million chains were analyzed. Missing linker regions with their corresponding sequences are identified from the “_pdbx_unobs_or_zero_occ_residues” entries in the mmCIF files and a total of 399,404 missing regions were recorded. For each missing region entry, the left and right observed amino acids are identified using the atomic coordinate data in the mmCIF files. For every linker region, following properties are derived (a) the end-to-end Ca-Ca distance between the two observed residues, measured in Å, (b) the length of the linker region, and (c) the sequence of the linker region. Lengths of these missing regions varied from 1 amino acid up to 3736 amino acids, and a sharp reduction of available PDB entries was observed upon increased length of linker sequence, with the trend visible in Supplementary Fig. 10E. To derive better collective properties, the files were grouped together by adaptively increasing the bin width of length of amino acids. For 1–50 amino acids, the bin width was kept at 1. For 50 onwards, the right bin-edge increases gradually, to 55, 60, 65, 70, 75, 100, 125, 150, 250 and 4000. From entries falling in every bin, the mean value of the Ca-Ca distances was calculated, and distance at quantiles varying from 0.0 to 1.0 in steps of 0.2. The analyzed data is presented in Supplementary Data 4. In Fig. 9A, the mean Ca-Ca distance, represented by black dots, was plotted against the length of the linker region. Distances at quantile level (0.0–1.0) was plotted as a color shade as annotated in the legend. For reference, a horizontal line (dotted blue) was plotted corresponding to 3.5 Å. Similarly, a line (solid blue) corresponding to 7 Å times the length of the missing region was plotted. The relation between the mean Ca-Ca distance (y) and the number of amino acids of the missing sequence (x) can be empirically characterized by a model function of the form: $y=A\left(1-{e}^{-{bx}}\right)+3.5$, where, constants A and b are observed to have values, 11 and 0.2, respectively. The model function was plotted as a red line. All plots related to PDB distance calculations (Fig. 9A, Supplementary Fig. 10D, E) were generated with the Pandas package in Python 3.9. The bubble plot containing all theoretical and experimental distance calculations (Supplementary Fig. 10C) was generated with the ggpubr 0.4.0 package in R.

Rotational displacement calculations of the *H. sapiens* E2o vertex trimer subunits vs. the experimentally resolved *C. thermophilum* E2o vertex trimer subunits (A, B, C) were performed as follows: first, *C. thermophilum* and *H. sapiens* (PDB ID: 6H05) E2o trimers were extracted and aligned upon subunit A in PyMol. Then, the “angle_between_domains” command was used to first calculate the angle between *C. thermophilum* subunit A and B. The same was done for the angle between *C. thermophilum* subunit A and *H. sapiens* subunit B. Then the values were subtracted. The same process was done for the subunit pair A and C, again keeping the rotation axis the same, aligned on subunit A. A visual representation of the measured domains can be seen in Supplementary Fig. 4A.

### Mass spectrometry protein identification and crosslinking mass spectrometry

Fractions 3–9 from the *C. thermophilum* native lysate fractionation described above were pooled into two pools (3–6 and 7–9). From each pool, 40 μL of sample was digested in-solution with trypsin as described previously^68,69^. To avoid protein precipitation during sample reduction and alkylation, 2 μL of 20% SDS were added. 1 μL of 200 mM DTT in 200 mM HEPES/NaOH pH 8.5 was added to reduce the protein samples, which were then incubated at 56 °C for 30 min. After reduction, alkylation followed with the addition of 2 μL of 400 mM chloroacetamide in 200 mM HEPES/NaOH, pH 8.5 and another incubation at 25 °C for 30 min. All excess chloroacetamide was quenched by adding 2 μL of 200 mM DTT in HEPES/NaOH, pH 8.5. After reduction and alkylation, the samples were used for single-pot solid-phase-enhanced sample preparation^68,69^. For this preparation, 5 μL of 10% v/v formic acid and 2 μL of Sera-Mag Beads were added, along with enough acetonitrile (ACN) to achieve a final ACN percentage of 50% v/v, then followed by an 8 min incubation and bead capture on a magnetic rack. The beads were then washed two times with the addition of 200 μL 70% ethanol and one more time with 200 μL of ACN. After resuspension in 10 μL of 0.8 μg of sequencing grade modified trypsin in 10 μL 100 mM HEPES/NaOH, pH 8.5, the beads were incubated overnight at 37 °C. The incubation was followed by a reverse phase cleanup step and then analyzed by liquid chromatography coupled to tandem mass spectrometry (LC-MS/MS) with a Q Exactive™ Plus Hybrid Quadrupole-Orbitrap™ Mass Spectrometer (ThermoFisher Scientific, USA). More specifically, an UltiMate™ 3000 RSLCnano System (ThermoFisher Scientific, USA) equipped with a trapping cartridge and an analytical column was used for peptide separation. For solvent A 0.1% v/v formic acid in LC-MS grade water and for solvent B 0.1% v/v formic acid in LC-MS grade CAN were used. All peptides were loaded onto the trapping cartridge with a solvent A set flow of 30 mL·min^−1^ for 3 min and eluted with a 0.3 mL·min^−1^ for 90 min of analysis time, starting with a 2–28% solvent B elution, then increased to 40% B, another 80% B washing step and finally re-equilibration to starting conditions. The LC system was directly coupled to the mass spectrometer using a Nanospray-Flex ion source and a Pico-Tip Emitter 360–μm OD × 20 μm ID; 10 μm tip. The mass spectrometer was operated in positive ion mode with a spray voltage of 2.3 kV and a capillary temperature of 275 °C. Full scan MS spectra with a mass range of 350–1400 m/z were acquired in profile mode using a resolution of 70,000 [maximum fill time of 100 ms or a maximum of 3e6 ions (automatic gain control, AGC)]. Fragmentation was triggered for the top 20 peaks with charge 2–4 on the MS scan (data-dependent acquisition) with a 20 s dynamic exclusion window (normalized collision energy was 26). Precursors were isolated with 1.7 m/z and MS/MS spectra were acquired in profile mode with a resolution of 17,500 (maximum fill time of 50 ms or an AGC target of 1e5 ions). For the data analysis, the MS raw data were analyzed by MaxQuant 1.6.1^70^. *Chaetomium thermophilum* proteomes sequences were downloaded from Uniprot with Proteome ID UP000008066. The MS data were searched against *Chaetomium thermophilum* proteomes sequences plus common contaminants sequence provided by MaxQuant. The default setting of MaxQuant was used with modification oxidation and acetyl (protein N-term). A false-discovery rate (FDR) cutoff of 1% was used for protein identification, and iBAQ intensity was used for label-free protein quantitation. When calculating iBAQ intensity, the maximum detector peak intensities of the peptide elution profile were used as the peptide intensity. Then, all identified peptide intensities were added and normalized by the total number of identified peptides.

For the preparation of the crosslinking mass spectrometry samples, a titration to identify the optimal crosslinker concentration was first performed (Supplementary Fig. 7). Fractions 3–9 from the *C. thermophilum* native lysate fractionation described above (Supplementary Fig. 7A) were pooled to a total volume of 1.4 mL and protein concentration of 0.48 mg·mL^−1^, then split into 7 parts of equal volume in each Eppendorf tube. In the first tube no crosslinking agent was added to be kept as control, while to the rest, 5 μL of 0.16, 0.32, 0.63, 1.25, 2.5 and 5 mM of the crosslinking agent BS_3_ was added respectively. The samples were incubated for 2 h on ice and the crosslinking reaction was then deactivated with the addition of 50 mM NH_4_HCO_3_, incubated again for 30 min on ice. The samples were then transferred in an acetone-compatible tube and 4 times the sample volume of cold (−20 °C) acetone was added, the tubes were vortexed, again incubated for 60 min at −20 °C and centrifuged for 10 min at 15,000 × *g*. The supernatant was properly removed and then the tubes were left open at RT in order for the acetone to evaporate. To visualize the titration results, each of the 7 tubes’ pellets were resuspended in 1X SDS-PAGE sample loading buffer (diluted from a 4X stock of 250 mM Tris–HCl pH 6.8, 8% w/v sodium dodecyl sulfate (SDS), 0.2% w/v bromophenol blue, 40% v/v glycerol, 20% v/v β-mercaptoethanol) to a final protein concentration of 10/20 μg·mL^−1^. The samples were then boiled for 5 min at 90 °C, loaded onto Mini-PROTEAN^®^ Precast Gels (BioRad, USA) and electrophorized for 60 min at 150 V. After the electrophoresis, gels were washed twice in water, stained with Coomassie staining solution and then destained until the background of the gel was fully destained. After visual inspection of gels, an optimal concentration of 1 mM BS_3_ was selected to proceed with sample crosslinking (Supplementary Fig. 7B). With optimal crosslinker concentration determined, fractions 3–9 from the *C. thermophilum* native lysate fractionation described above were pooled again, but this time in two pools, 3–6 and 7–9. In a Protein LoBind Eppendorf tube for each pool, 100 μL of 8 M urea was added and then centrifuged overnight in a centrifugal vacuum evaporator at RT. 100 μL from each pool was then transferred to each of the tubes containing urea powder and 100 mM of ammonium bicarbonate (ABC) was added. DTT was then also added to a final concentration of 2.5 mM (from a 100 mM DDT stock dissolved in 100 mM ABC) and incubated for 30 min at RT. After the incubation was over, 2-Iodoacetamide (IAA) was added to a final concentration of 5 mM followed by incubation for 30 min, at RT in the dark. The reaction was quenched by the addition of 2.5 mM DTT to prevent modification of serine in the trypsin active sites. LysC (1 μg μL^−1^ stock, in 1:100 ratio, w/w) was subsequently added and samples were again incubated at RT for 4.5 h. An addition of 50 mM ABC to the sample reduced the urea concentration to <2 M. Trypsin (from 1 μg μL^−1^ stock, in 1:50 ratio, w/w) was introduced to the samples which were then incubated overnight at RT, followed by STop And Go Extraction (STAGE) TIPS desalting procedure, as described in ref. ^71^. The final crosslinked peptide samples were subjected to the same LC-MS/MS process and the results were analyzed as described above in the MS protein identification method. All plots, visualizing MS/XL-MS data were created with the xiVIEW online webserver^72^.

### Network analysis and community identification

Identified proteins from the mass spectrometry were mapped in the provided higher-order assemblies described in the Kastritis et al. (2017)^19^. These higher-order assemblies were further enriched utilizing homology search per Uniprot entry against the Protein Data Bank, to also include possible homologous subunits resolved in PDB structures after 2017. For this, Blastp was used (https://blast.ncbi.nlm.nih.gov/Blast.cgi?PAGE=Proteins) by default against the PDB database. Next, networks and communities provided in Kastritis et al. (2017) acted as a basis for further expanding networks reported in this study. This was performed by mapping the proteins to those networks, including them in the STRING^73^ database and expanding the identified interactions into a network with first- and second- shell interactors and communities. Then, *C. thermophilum*-specific local STRING network clusters were retrieved and coverage per string cluster is reported by simple division of identified proteins over all proteins present in the STRING cluster. This was performed from these expanded networks via back-mapping to the MS and XL-MS data. Finally, networks were checked for biological significance utilizing the in-database enrichment detection criterion^74^, showing that all 54 clusters (protein communities) derived here were significantly enriched in biological interactions. Visualizing of the networks was performed with Cytoscape^75^. Recovery of the complete TCA cycle was inferred utilizing KEGG^76^.

### Multiple sequence alignments and additional validation of AlphaFold2-derived models with crosslinking data

Uniprot ID: G0S3G5 (*C. thermophilum* putative holocytochrome C synthase - sequence length: 382), Uniprot ID: Q7S3Z3 (characterized in^13^ as the *N. crassa* Kgd4 protein - sequence length: 130), along with Uniprot ID: Q7S3Z2 (*N. crassa* putative holocytochrome C synthase - sequence length: 317) were downloaded in.fasta format from the Uniprot database and aligned with Clustal Omega^77^ in two separate alignment pairs: G0S3G5 with Q7S3Z3 and G0S3G5 with Q7S3Z2 separately. The.aln files were downloaded and visualized with Jalview^78^. Alignment results are displayed in Supplementary Fig. 8. The protein IDs in Uniprot have been updated, with P9WES7 now corresponding to the putative holocytochrome C synthase (length: 287 a.a.) and P9WES8 to the identified E3BPo (length: 135 a.a.).

AlphaFold2-derived models for all proteins involved in the formation of the OGDHc metabolon, namely E1o, E2o and E3 were validated for their quality by employing the intra-molecular cross-links derived from the cross-linking analysis performed above. All identified intra-molecular cross-links of E1o, E2o and E3, respectively, were mapped onto the corresponding models via the use of an in-house python script and manually assessed for their feasibility. Distance distributions were plotted for each model in Supplementary Fig. 9A, and the corresponding data is included in Supplementary Data 2.

### Statistics and reproducibility

All statistical analysis performed is appropriately described in each of the Methods subsections and includes statistical methods applied for the analysis, confidence intervals, number of biological replicates, number of technical replicates, standard deviations and cross-correlations scores.

### Reporting summary

Further information on research design is available in the Nature Portfolio Reporting Summary linked to this article.

## Supplementary information

Supplementary Information

Description of Additional Supplementary Files

Supplementary Data 1

Supplementary Data 2

Supplementary Data 3

Supplementary Data 4

Supplementary Data 5

Supplementary Data 6

Reporting Summary

## Data Availability

The data that support this study are available from the corresponding author upon reasonable request. Source data for all figures is provided with this paper in the corresponding Supplementary Data items. Cryo-EM map and model for the native 24-mer core of the OGDHc has been deposited in EMDB and PDB under accession numbers EMD-16900 and 8OIU respectively. Asymmetric cryo-EM map of the OGDHc is included in EMD-16900. Raw movies, summed micrographs, and grid atlases have been deposited in EMPIAR under accession number EMPIAR-11502. Mass spectrometry data is available as Supplementary Data 2.
