# Supplementary material for: Structural analysis of an endogenous 4-megadalton succinyl-CoA-generating metabolon
Source: Commun Biol. 2023 May 22;6:552. doi: 10.1038/s42003-023-04885-0 (PMC10203282; doi:10.1038/s42003-023-04885-0)
Supplement: Supplementary file 1 — Supplementary Information [file 42003_2023_4885_MOESM1_ESM.pdf]

Supplementary Information

Structural analysis of an endogenous 4-megadalton succinyl-coA-generating metabolon

Ioannis Skalidis[1,2,#], Fotis L. Kyrilis[1,2,#], Christian Tüting[1,#], Farzad Hamdi[1], Toni K. Träger[1,2], Jaydeep Belapure[1], Gerd Hause[3], Marta Fratini[4], Francis J. O’Reilly[5], Ingo Heilmann[4], Juri Rappsilber[6,7], Panagiotis L. Kastritis[1,2,3,8] *.

[1]Interdisciplinary Research Center HALOmem, Charles Tanford Protein Center, Martin Luther University Halle-Wittenberg, Kurt-Mothes-Straße 3a, 06120 Halle/Saale, Germany

[2]Institute of Biochemistry and Biotechnology, Martin Luther University Halle-Wittenberg, Kurt-Mothes-Straße 3, 06120 Halle/Saale, Germany

[3]Biozentrum, Martin Luther University Halle-Wittenberg, Weinbergweg 22, 06120 Halle/Saale, Germany

[4]Department of Plant Biochemistry, Institute of Biochemistry and Biotechnology, Martin Luther University Halle-Wittenberg, Kurt-Mothes-Str. 3a, 06120 Halle/Saale, Germany

[5]Center for Structural Biology, Center for Cancer Research, National Cancer Institute (NCI), Frederick, MD 21702-1201, USA

[6]Bioanalytics, Institute of Biotechnology, Technische Universität Berlin, 13355 Berlin, Germany

[7]Wellcome Centre for Cell Biology, School of Biological Sciences, University of Edinburgh, Edinburgh EH9 3BF, Scotland, United Kingdom

[8] Institute of Chemical Biology, National Hellenic Research Foundation, Athens 11635, Greece

[#]These authors contributed equally.

*Corresponding author.

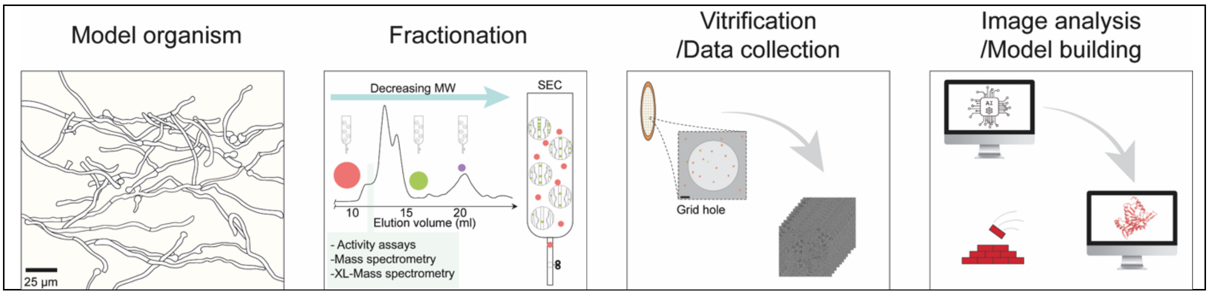

<u>Supplementary Figure 1</u>: **From organism to structure.** Schematic representation of the workflow that is followed in order to produce the native cell extract and characterize it.

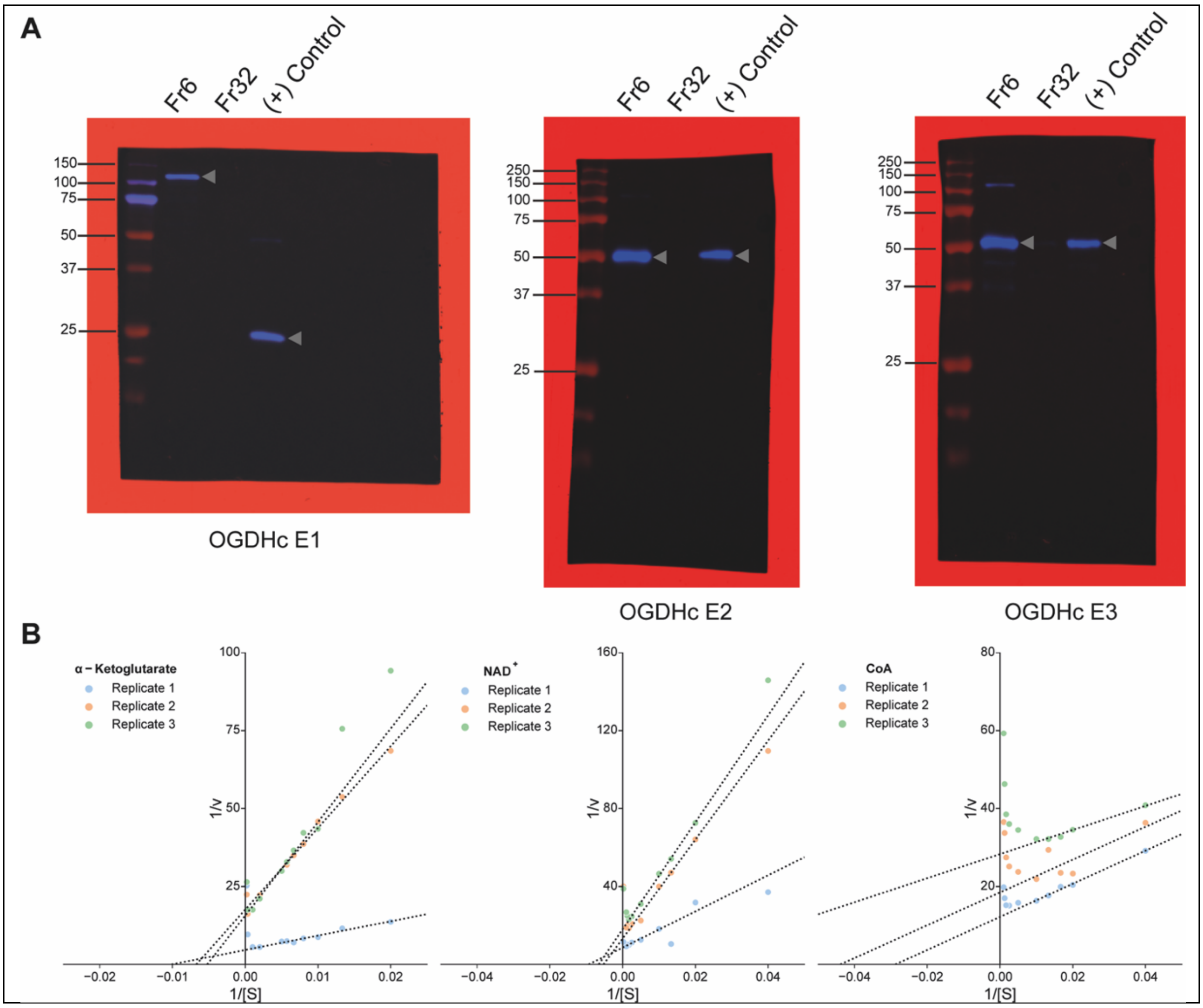

Supplementary Figure 2: **Uncropped WB gels and Lineweaver-Burk plots. (A)** Uncropped WB gels for each of the OGDHc components. Relevant bands have been annotated with a gray arrow. For a positive control of E1o detection, the shorter peptide that was produced as an antigen in order to generate the antibody was employed. **(B)** Lineweaver-Burk plots for each of the substrates that are involved in the OGDHc reaction, showing the values for the 3 biological replicates for each.

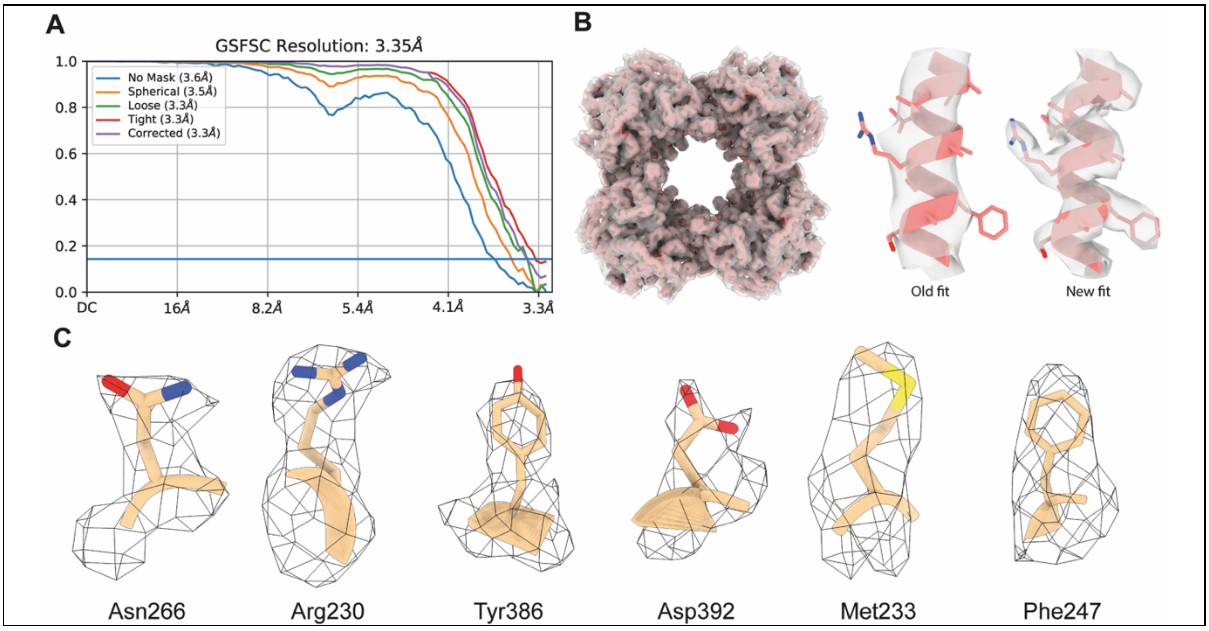

<u>Supplementary Figure 3</u>: **FSC plot, improvement over previously published reconstructions of the E2o 24-meric *C. thermophilum* core and side-chain density resolution examples. (A)** Gold-standard (0.143) FSC plot for the E2o core map. **(B)** Comparison between the cell extract-derived and the previously published OGDHc E2o core map (EMD-13844). On the left, the cell extract-derived E2o core map (salmon) is fit into the previously derived E2o core map (EMD-13844, gray). On the right, the marked improvement in side-chain density coverage between the old and new maps can be observed. **(C)** Various examples taken from the cryo-EM resolved fitted model, showing confident placement of side-chains in their corresponding densities.

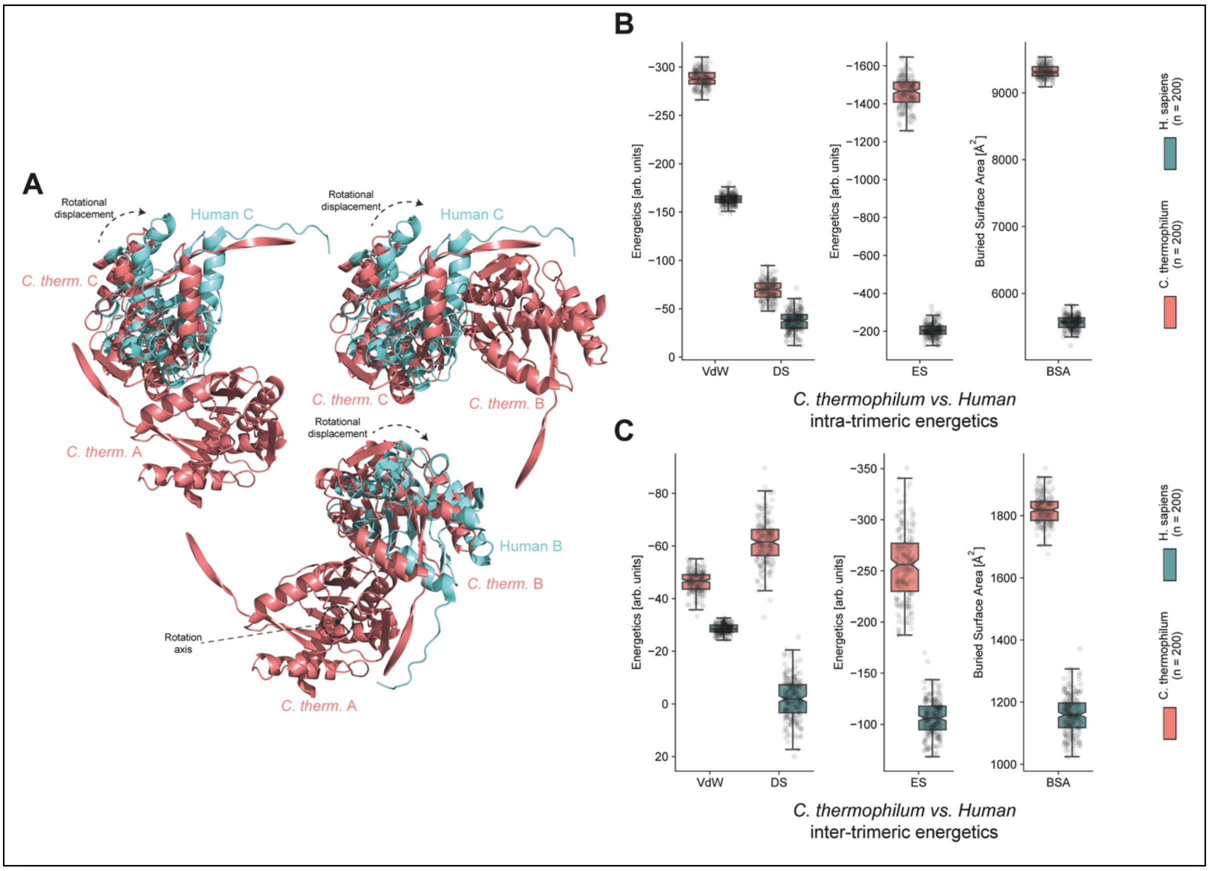

Supplementary Figure 4: **Rotational displacement calculations, interfaces of LD with all OGDHc components and comparative energetics between *C. thermophilum* E2o interfaces and its mesophilic human counterpart. (A)** Models representing how the rotational displacement calculations were performed, between the E2o core vertex trimer of *C. thermophilum* and Human. **(B)** *C. thermophilum* and Human E2o core intra-trimeric energetics display stronger forces that contribute to the *C. thermophilum* E2o core compaction. **(C)** *C. thermophilum* and Human E2o core inter-trimeric energetics display stronger forces that contribute to the *C. thermophilum* E2o core compaction. Original values for plotting can be found in **Supplementary Data 5**.

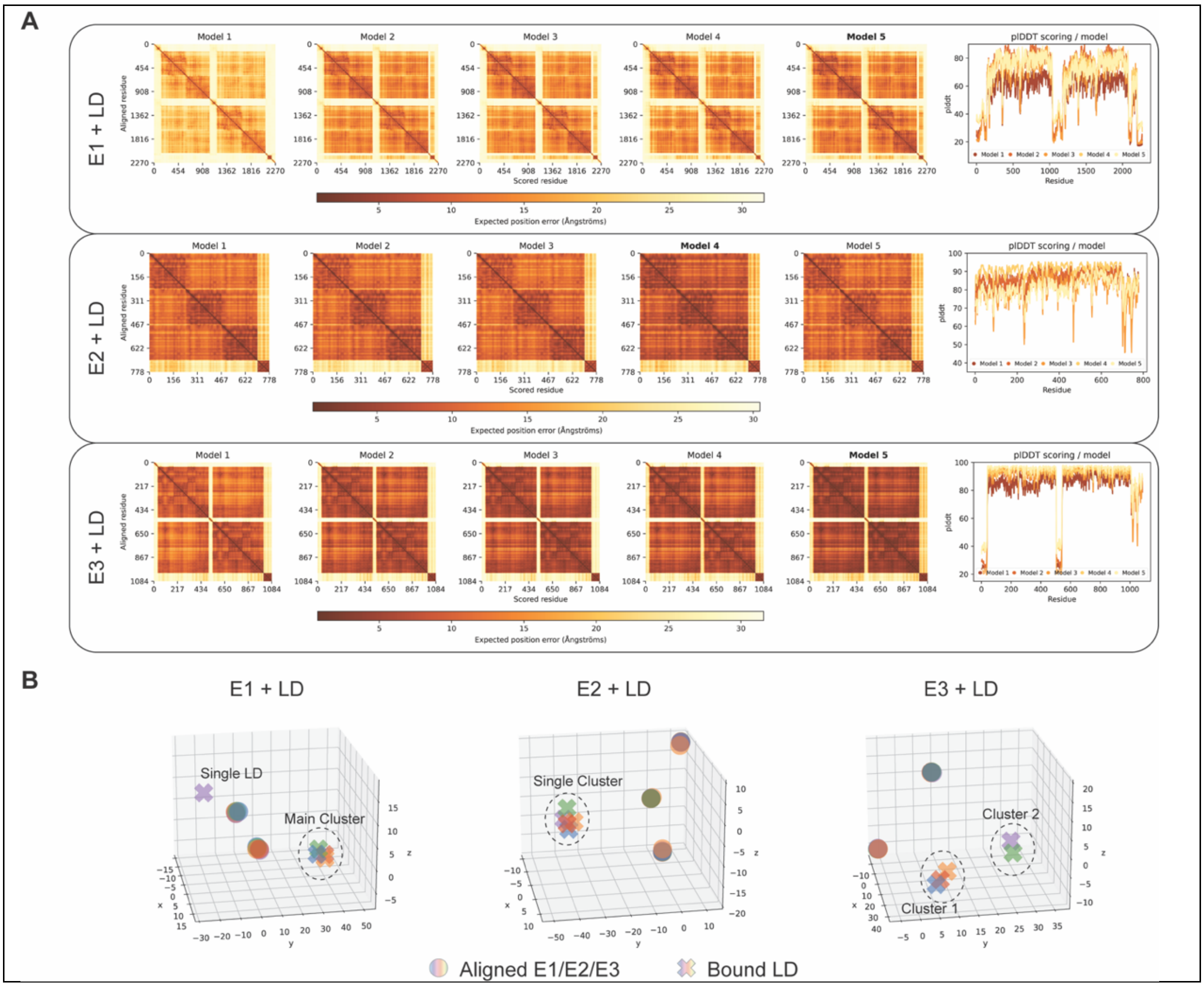

Supplementary Figure 5: **AlphaFold2 model validation and E3BPo sequence alignments. (A)** Positional Alignment Error plots and plDDT scoring for the top 5 models of each AlphaFold-multimer prediction of E1o, E2o and E3 in complex with the E2o LD domain. **(B)** Spatial plots showing the position of the LD predicted for each solution returned by AlphaFold-multimer. A single cluster is observed for the predicted interaction between E1o-LD and E2o-LD, whereas 2 clusters can be seen for the E3-LD interaction.

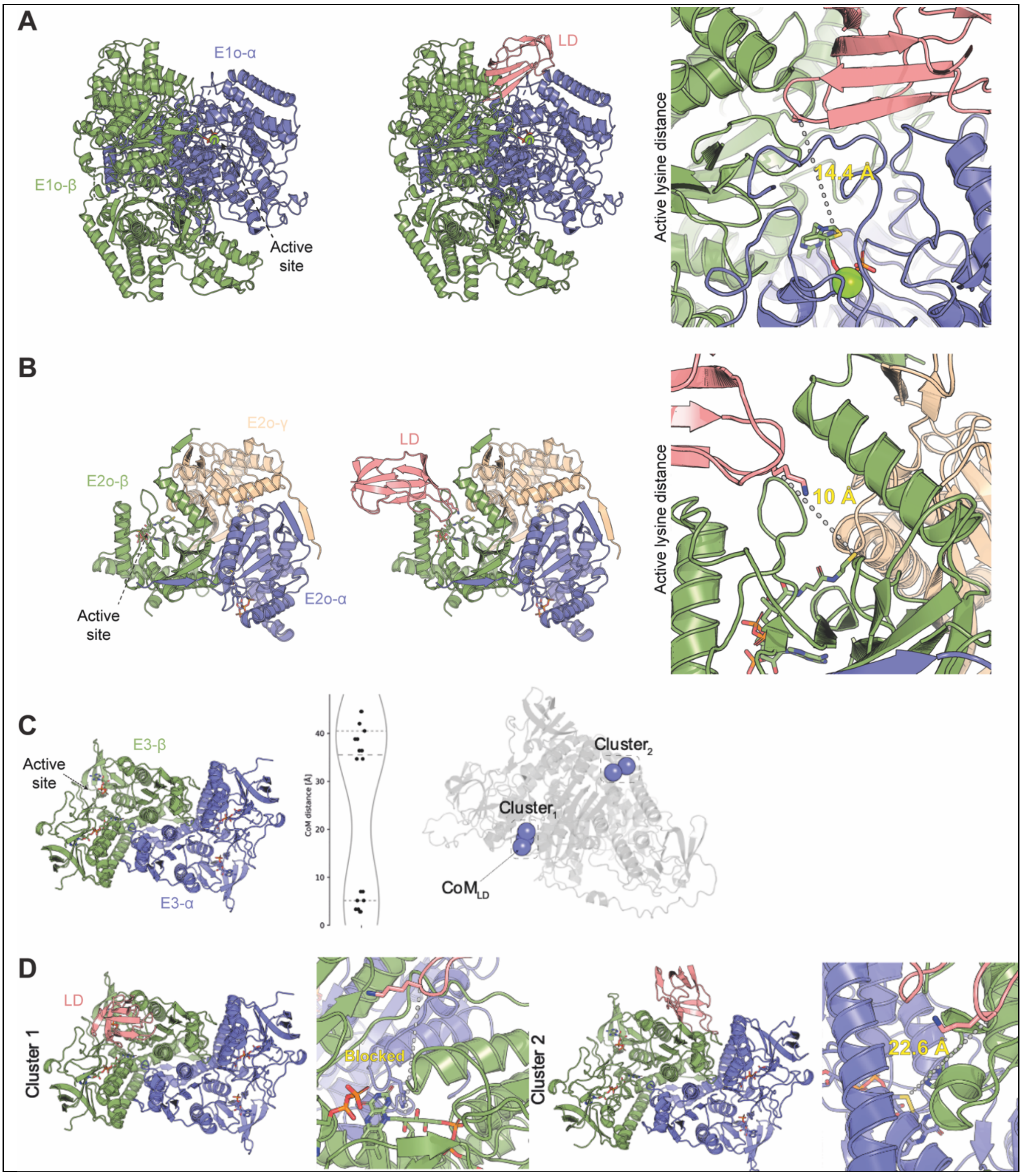

Supplementary Figure 6: **AlphaFold2 predicted models of the OGDHc subunits in complex with the E2o LD domain. (A)** AlphaFold2 predicts the LD as bound in the dimeric interface. The lipoylated lysine is in close distance (14.4 Å) to the C2 atom of the ThDP, which is succinylated during decarboxylation of α-ketoglutarate in the first step of the reaction of OGHDc. The binding cavity of the generated AlphaFold2 model is not sterically blocked, indicating a plausible docking solution. **(B)** In each dimeric interaction interface in these trimeric E2o building blocks, a CoA binding site is present. Clustering of all AF solutions showed all LDs within a distance range of 10 Å, indicating a single prediction solution. The LD domain is bound to a monomeric E2, meaning that theoretically 24 LD domains could be bound by the OGHDc core simultaneously. The localization of the lipoyl-lysine is close to the CoA binding site, with a distance between the Cα atom of the lysine and the thiol group of CoA, where

the succinate from the lipoate is transferred to, of 13.7 Å. The binding cavity is accessible, indicating, again, a plausible docking solution. **(C)** In the predicted structures of E3 with bound LD, there are two clear cluster (n = 3 and n = 2), with clear differences in the localization of the LD. **(D)** Inspecting the two different clusters of the bound LD, in the first cluster, the LD is located at a monomer near the NAD+ binding site, whereas in the second cluster, the LD is bound in the dimeric interface. Mapping the reaction path, in cluster 1, the reaction path is blocked by the FAD, whereas in the second cluster, the lipoylated lysine are in reasonable distance to the disulfide bond in the active side, with a distance of 22.6 Å.

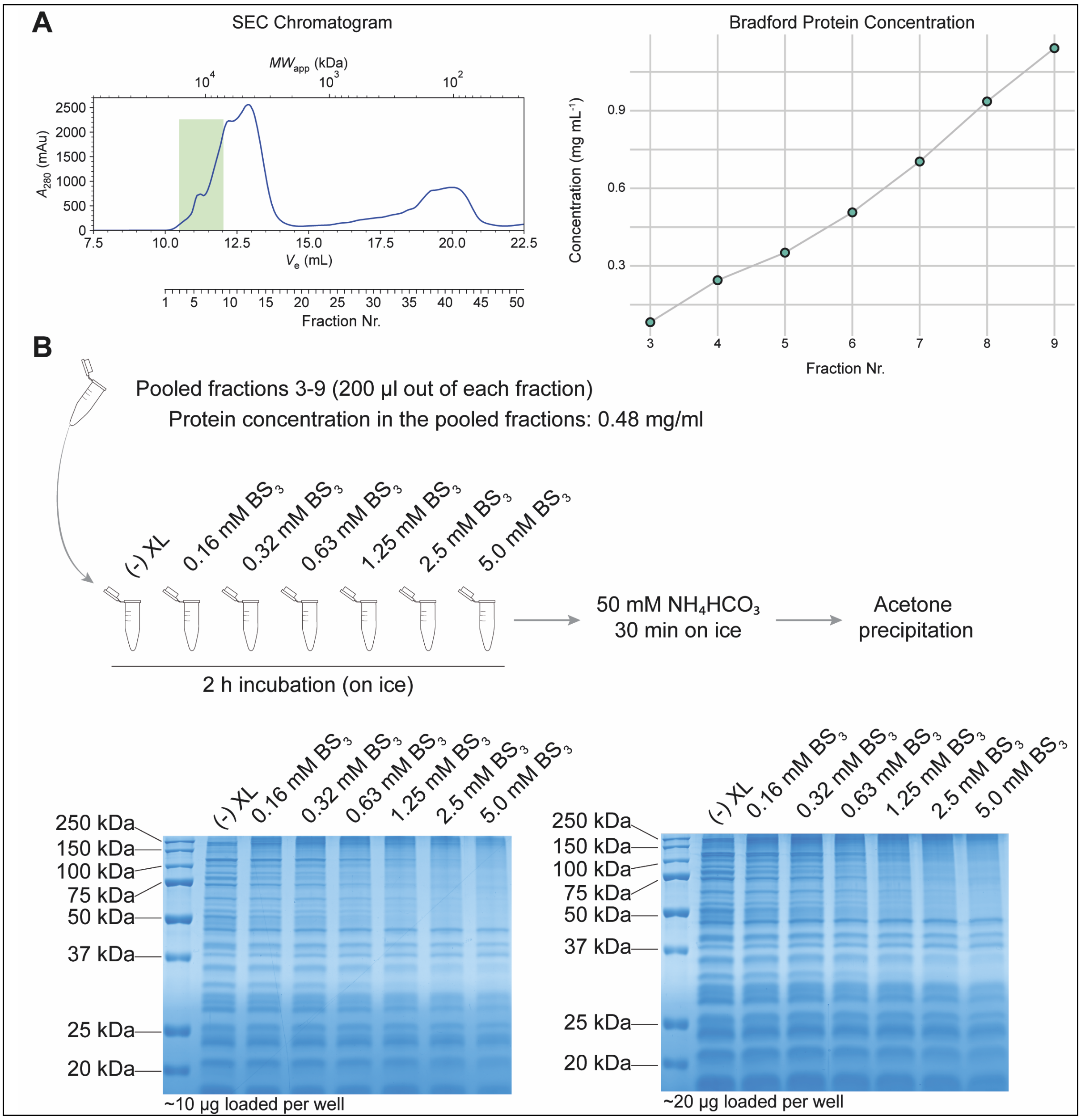

Supplementary Figure 7: **Cross-linker benchmarking**. **(A)** Sample preparation for cross-linking experiments. The chromatogram of the SEC fractionation of the native *C. thermophilum* lysate that was performed is visible on the top, with the fractions that were pooled for the benchmarking annotated with a green box. On the right, protein concentrations for each of the fractions 3-9 are shown. **(B)** Schematic representation of the crosslinking process. The gels that were used to validate the crosslinking process can be seen on the bottom.

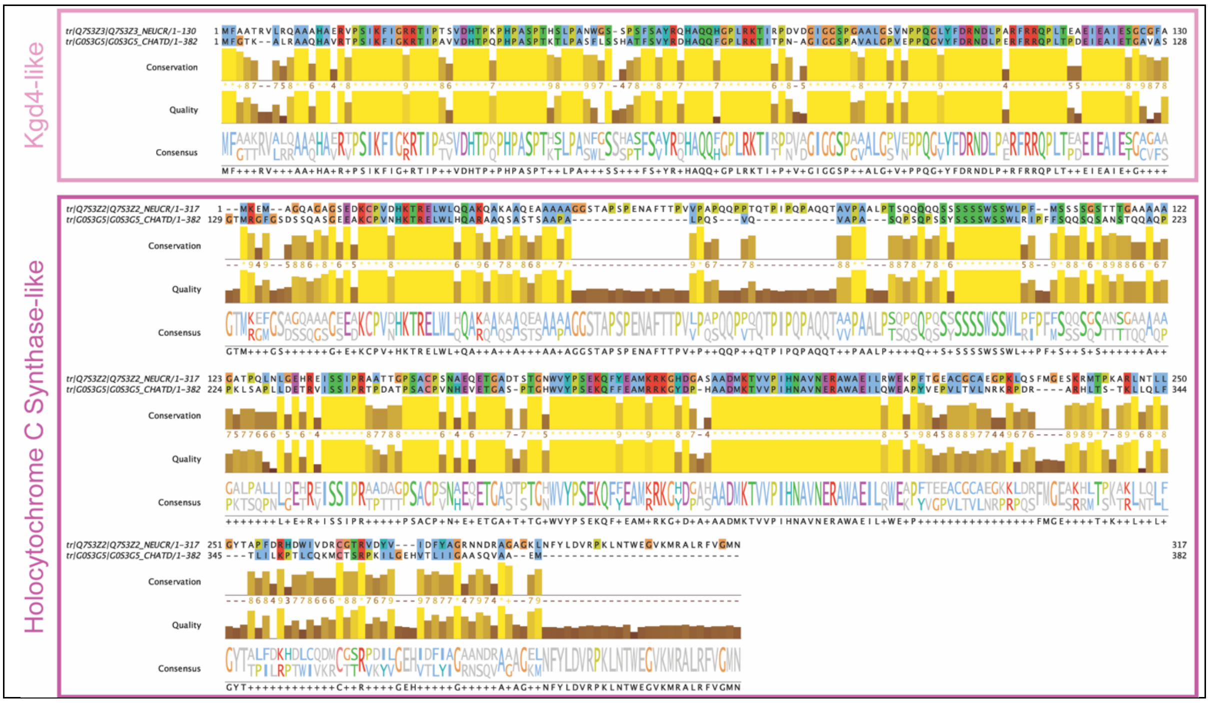

Supplementary Figure 8: **Alignments for the identification of E3BPo.** Sequence alignment of *N. crassa* KGD4 and HCCS to the *C. thermophilum* putative HCCS-annotated protein sequence. The *N. crassa* KGD4 protein sequence aligns with high confidence to the first 129 residues of the *C. thermophilum* sequence, while the *N. crassa* HCCS aligns to the rest 254 residues, strongly indicating that the *C. thermophilum* protein sequence is wrongly annotated and is in reality two separate proteins.

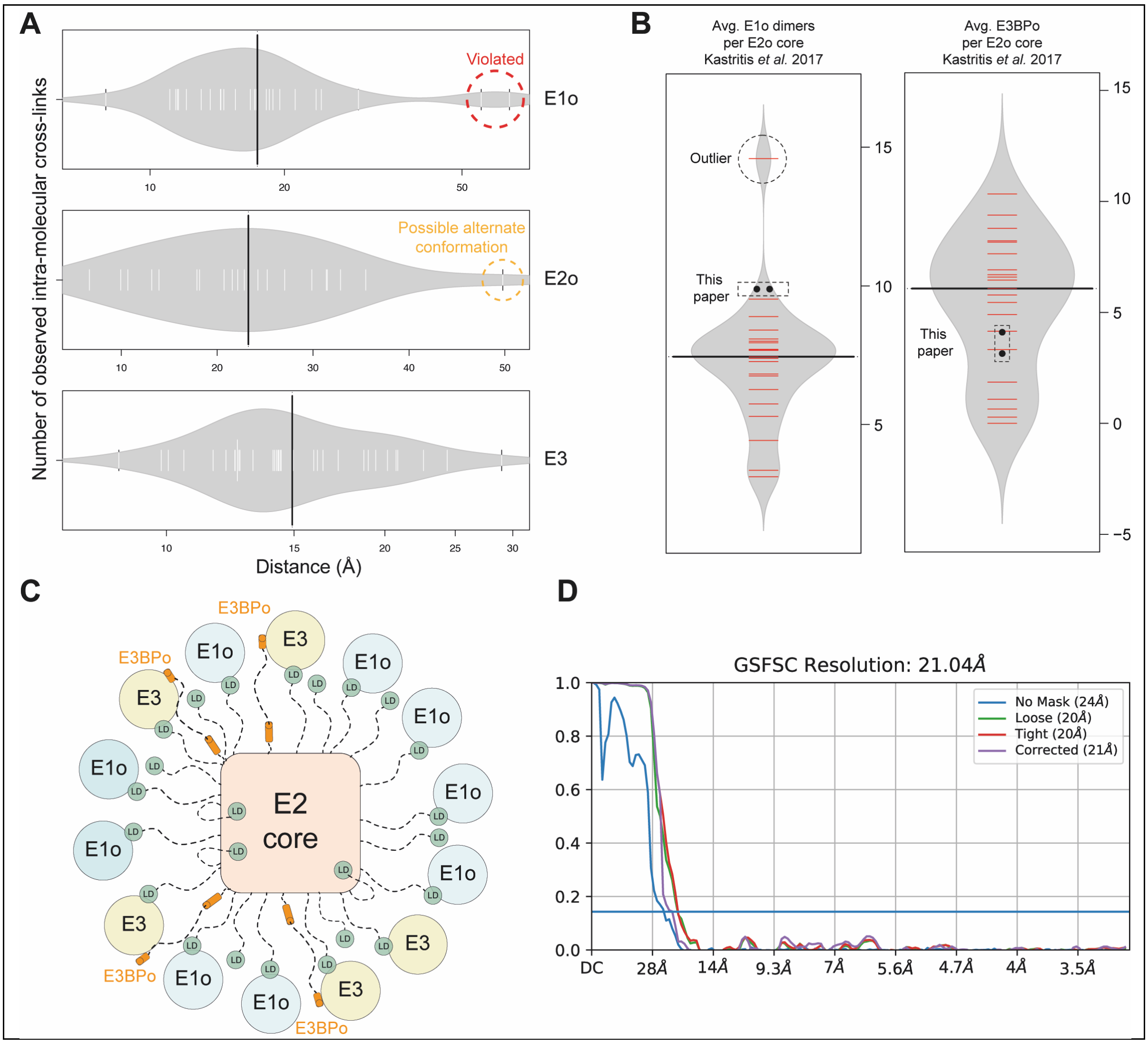

<u>*Supplementary Figure 9*</u>: **Cross-linking validation of OGDHc components, MS-derived stoichiometric calculations, and FSC for the full OGDHc complex. (A)** Plots depicting the distribution of residue distances in the AlphaFold2-derived models as captured by the cross-linking experiments. In E1o (top), two cross-links violate the expected maximum distance of the cross-linker (~35 Å, BS_3, red circle), and in E2o violating cross-links may be attributed to different conformations of the flexible lipoyl-arm (middle, orange circle). **(B)** Stoichiometric calculations of previously published MS data (Kastritis *et al.,* 2017) and newly derived MS data reveal the stoichiometry of the E1o and E3BPo components that take part in the formation of the fully active, native OGDHc. **(C)** Schematic representation based on the stoichiometries derived from the MS data presented in this article. The 24-mer E2o core is surrounded by at least 4 E3 dimers that are bound to 4 E3BPo, joined by 9 E1o dimers. **(D)** Gold-standard (0.143) FSC plot for the OGDHc map.

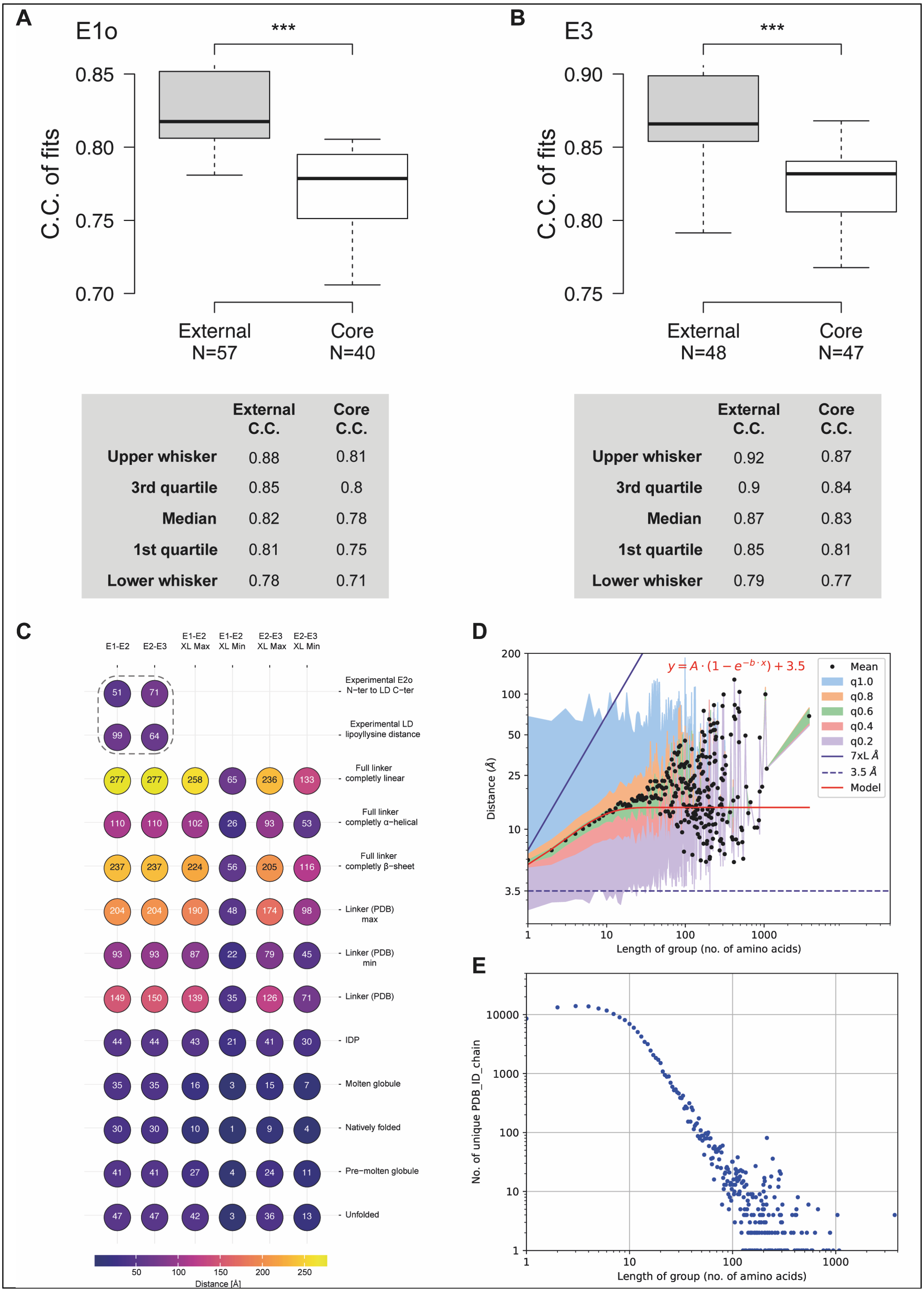

	External C.C.	Core C.C.
**Upper whisker**	0.88	0.81
**3rd quartile**	0.85	0.8
**Median**	0.82	0.78
**1st quartile**	0.81	0.75
**Lower whisker**	0.78	0.71

	External C.C.	Core C.C.
**Upper whisker**	0.92	0.87
**3rd quartile**	0.9	0.84
**Median**	0.87	0.83
**1st quartile**	0.85	0.81
**Lower whisker**	0.79	0.77

Supplementary Figure 10: **Cross-correlation of fits, experimental and theoretical linker E2o distances, PDB linker distances. (A)** Boxplot of cross-correlation values of E1o fits in the OGDHc map. The E1o dimer fits to the external map densities with

statistical significance compared to the internal densities. **(B)** Boxplot of cross-correlation values of E3 fits in the OGDHc map. The E3 dimer fits to the external map densities with statistical significance compared to the internal densities. **(C)** Bubble plot of all experimental and theoretical E2o linker distance values. XL-min and XL-max annotate the distance from the first *N-ter* residue of the E2o that is resolved in the core map to the closest and farthest cross-linked lysine to a peripheral subunit respectively. **(D)** Graph representing the mean distance values of all unresolved amino-acid sequences belonging to all protein structures deposited in the PDB. Black dots represent the mean value of a length of amino-acids group, with different colors the standard deviation after the integration of each quartile of total data, as denoted in the plot legend. The red line represents a fitted model that describes the relationship between the distance and the length of amino-acid groups. The blue line represents the 2x theoretical distance of an amino-acid sequence, while the dashed blue line represents the theoretical lower limit of any amino-acid sequence. **(E)** Plot representing the trend between the number of PDB structures incorporating a certain sequence length of unresolved amino-acids.

	OGDHc E2o core (EMD-16900) (PDB 8OIU)	OGDH complex (Map included in EMD-16900)
**Data collection and processing**		
Magnification	92000X	92000X
Voltage (kV)	200	200
Microscope model	TFS Glacios	TFS Glacios
Camera model	TFS Falcon IIIEC	TFS Falcon IIIEC
Number of frames	13	13
Electron exposure (e–/Å^2)	30	30
Per-frame exposure (e–/Å^2)	2.3	2.3
Defocus range (μm)	-0.6 to -2.0	-0.6 to -2.0
Pixel size (Å)	1.5678	1.5678
Images acquired (no.)	25803	25803
Acquisition software	TFS EPU 2	TFS EPU 2
Symmetry imposed	O	C1
Initial particle images (no.)	3,596,302	2,891,518
Final particle images (no.)	52,034	5,178
Map resolution (Å)	3.35	21.04
FSC threshold	0.143	0.143
Map B-factor	176.9	-
**Refinement**		
Initial model used (PDB code)	7Q5Q	
Map sharpening *B* factor (Å^2)	0 (not modified)	
Model composition		
Non-hydrogen atoms	1812	
Protein residues	233	
B factors (Å^2)		
Protein (min/max/mean)	7.48/59.64/22.96	
R.m.s. deviations		
Bond lengths (Å)	0.005	
Bond angles (°)	1.134	
Validation		
MolProbity score	2.61	
Clashscore	16.72	

Poor rotamers (%)	3.05
Ramachandran plot	
Favored (%)	90.91
Allowed (%)	9.09
Disallowed (%)	0.00
Map-CC	0.79

Supplementary Table 1: **OGDHc E2o core and complex acquisition and reconstruction parameters.**
