# Supplementary material for: Structural analysis of an endogenous 4-megadalton succinyl-CoA-generating metabolon
Source: Commun Biol. 2023 May 22;6:552. doi: 10.1038/s42003-023-04885-0 (PMC10203282; doi:10.1038/s42003-023-04885-0)
Supplement: Supplementary file 2 — Description of Additional Supplementary Files [file 42003_2023_4885_MOESM2_ESM.docx]

**Description of Additional Supplementary Files**

**File name:** Supplementary Data 1

**Description:** All values plotted for the kinetic characterization of the OGDHc component reactions.

**File name:** Supplementary Data 2

**Description:** All values related to XL-MS, MS identification, in-fraction community annotation, stoichiometric calculations and intra-molecular crosslinking validation of AlphaFold2-derived models.

**File name:** Supplementary Data 3

**Description:** All values and statistics related to OGDHc peripheral subunit fits and distance calculations.

**File name:** Supplementary Data 4

**Description:** All values related to plots of unresolved amino-acid sequences in all PDB entries.

**File name:** Supplementary Data 5

**Description:** All values reported in the HADDOCK scoring plots.

**File name:** Supplementary Data 6

**Description:** All residue frequencies of residues participating in the interface between E1o, E3 and the E2o LD domain.
